# Integration of metabolomics and transcriptomics unravels the identification of *TPS* gene family and functional characterization of a sesquiterpenoid synthesis gene in *Curcuma kwangsiensis*

**DOI:** 10.3389/fpls.2025.1703946

**Published:** 2025-12-03

**Authors:** Ruhong Ming, Xin Xie, Wei Ling, Yuping Wei, Jianhua Chen, Shaochang Yao, Yong Tan, Liangbo Li, Rongshao Huang, Ding Huang, Jian Xiao

**Affiliations:** 1 University Engineering Research Center of Characteristic Traditional Chinese Medicine and Ethnomedicine, Guangxi University of Chinese Medicine, Nanning, China; 2 College of Pharmacy, Guangxi University of Chinese Medicine, Nanning, China; 3 Guangxi Engineering Research Center for High-Value Utilization of Guangxi-Produced Authentic medicinal Herbs, Guangxi University of Chinese Medicine, Nanning, China

**Keywords:** *Curcuma kwangsiensis*, sesquiterpenes, terpene synthase, transcriptome, metabolome

## Abstract

**Introduction:**

*Curcuma kwangsiensis* S.G. Lee et C.F. Liang, a member of the *Zingiberaceae* family, is abundant in sesquiterpenes. However, the sesquiterpene metabolome of *C. kwangsiensis* remains poorly characterized, and its terpene synthase (*TPS*) gene family has not yet been identified.

**Methods:**

In this study, metabolomics analysis was employed to systematically profile the metabolites in different tissues of *C. kwangsiensis* and identify differential expressed metabolites. Transcriptome sequencing technology was utilized to analyze the different expressed genes (DEGs), providing insights into the molecular basis of its secondary metabolism.

**Results:**

The experimental results demonstrated that a total of 177 terpenoids were upregulated in the rhizome, while 175 terpenoids showed upregulation in the tuber. KEGG classification revealed that nine differential metabolites were identified in the Sesquiterpenoid and Triterpenoid Biosynthesis pathway, of which eight were sesquiterpenes. By employing bioinformatics approaches to identify the *TPS* gene family in *C. kwangsiensis*, a total of 24 *TPS* gene family members were identified. One candidate gene *CkTPS10* was cloned, heterologously expressed in *Saccharomyces cerevisiae*, and functionally characterized. The catalytic products, α-copaene and farnesol, of the enzyme were consistent with the results of key differential metabolite screening, indicating that the *CkTPS10* play a pivotal role in the biosynthesis of sesquiterpene components in *C. kwangsiensis*.

**Conclusion and Discussion:**

Integrated transcriptomic and metabolomic analysis represents an efficient approach for investigating the secondary metabolites of *C. kwangsiensis*, offering a theoretical foundation for deciphering the biosynthetic pathway of sesquiterpene compounds in this plant.

## 1 Introduction

*Curcuma kwangsiensis*, commonly known as *Curcuma phaeocaulis* Valeton, is a key botanical source of the traditional Chinese medicine Curcuma. The dried roots including rhizome and tuber of *C.kwangsiensis* are commonly used in traditional Chinese medicine for promoting Qi circulation, dissipating blood stasis, resolving stagnation, and alleviating pain. In the modern world, it is commonly used to treat various conditions, including masses, lumps, blood stasis-induced menstrual disorders, chest congestion, cardiac pain, and abdominal distension with pain (Zhou et al., 2016; Zhu et al., 2023). The Curcuma contains bioactive chemical constituents, and its major bioactive components are curcuminoids and sesquiterpenoids from volatile oils, such as curcumol, curgerenone, β-elemene, germacrone, and curdione (Liu et al., 2013; Li et al., 2017; Fu et al., 2025).

As the most abundant class of plant metabolites with the greatest structural diversity, terpenes exert vital functions in various facets of how plants grow and develop (Yu et al., 2018). Terpenes are isoprene polymers derived from five-carbon building blocks, isopentenyl pyrophosphate (IPP) and dimethylallyl pyrophosphate (DMAPP). Biosynthesized via the plastidic methylerythritol phosphate (MEP) or cytosolic mevalonic acid (MVA) pathways, these compounds are catalyzed by terpene synthases (*TPS*) to form diverse classes, including monoterpenes (C10), sesquiterpenes (C15), and diterpenes (C20), among others (Huang et al., 2021). As a crucial enzyme in the downstream synthesis of terpenoid compounds, *TPS* genes are categorized into seven subfamilies: *TPS-a*, *TPS-b*, *TPS-c*, *TPS-d*, *TPS-e/f*, *TPS-g*, and *TPS-h*. Among them, the *TPS-a*, *TPS-b* and *TPS-g* is a subfamily unique to angiosperms, while *TPS-d* is only found in gymnosperms. The subfamilies *TPS-c* and *TPS-e/f* are present in both angiosperms and gymnosperms, while the *TPS-h* subfamily has been exclusively identified in *Selaginella moellendorffii* (Chen et al., 2011; Jia et al., 2016). Protein sequence analysis indicates that sesquiterpene synthases (*STPSs*) and diterpene synthases (*DTPSs*) in angiosperms are classified under the *TPS-a* subfamily (Chen et al., 2011; Das et al., 2021). To date, only a limited number of *TPS* genes have been cloned and functionally characterized in the genus Curcuma in the *Zingiberaceae* family species (Sun et al., 2019).

Due to the remarkable therapeutic effects of sesquiterpenoid compounds from *C. kwangsiensis* in treating various diseases (Gao et al., 2021; Wang et al., 2018; Zeng et al., 2012; Yuan et al., 2022)research on these compounds has emerged as a prominent focus in recent years. The analysis of sesquiterpenoid components from *C. kwangsiensis* has become increasingly sophisticated, and investigations into their pharmacological effects have deepened and expanded. However, significant gaps remain in the understanding of biosynthetic pathways for these sesquiterpenoids, with key enzymes in the terpenoid biosynthesis cascade still awaiting exploration and identification. Based on the above, this study aims to identify the terpene synthase gene family in *C. kwangsiensis* using transcriptomic and metabolomic approaches, and systematically characterize the key putative enzymes in the sesquiterpenoids biosynthesis metabolic pathway from *C. kwangsiensis*. The research will excavate key genes involved in the biosynthetic pathway of sesquiterpenoid compounds from this plant and perform functional validation, with the objective of mining genetic information from both intrinsic factors and phenotypic expressions.

## 2 Materials and methods

### 2.1 Plant material

The experimental materials of *Curcuma kwangsiensis* S. G. Lee were obtained from the Xianhu Pharmaceutical Nursery of Guangxi University of Chinese Medicine (22°48’5”N, 108°29’49”E) (**Supplementary Figure S1**). The original plants and their macroscopic characteristics were identified by Associate Professor Li Bin from the Department of Medicinal Plants of Guangxi University of Chinese Medicine. Leaves, rhizomes, and tubers of *C. kwangsiensis* were collected, and immediately frozen and stored at -80 °C for subsequent analyses, with three biological replicates established for each tissue.

### 2.2 Sample extraction and HS-SPME-GC-MS analysis

The leaves (L), rhizomes (EZ or GJ, termed “E Zhu”), and tuberous roots (YJ, termed “Yu Jin”) of *C. kwangsiensis* were ground in liquid nitrogen, homogenized by vortex mixing, and around 500 mg of each homogenized sample was weighed and placed into a headspace vial. Each sample then received a saturated NaCl solution along with 10 μL of internal standard solution (50 μg/mL, 3-Hexanone-2,2,4,4-d4). Sample extraction was performed using automated headspace solid-phase microextraction (HS-SPME).

Extraction conditions were set as follows: samples were shaken at a constant temperature of 60 °C for 5 min. A 120-µm DVB/CWR/PDMS extraction head was inserted into the headspace vial containing the sample, followed by headspace extraction for 15 min, desorbed for 5 min at 250°C. Separation and identification were performed using a GC-MS system (Agilent 8890-7000D) equipped with a DB-5MS capillary column (30 m × 0.25 mm × 0.25 µm, Agilent J&W Scientific, Folsom, CA, USA).

The mass spectrometry conditions were set as follows: electron ionization (EI) ion source at 230°C, quadrupole temperature at 150°C, mass spectrometry interface temperature at 280°C, electron energy of 70 eV, operated in selected ion monitoring (SIM) scan mode, and subjected to accurate scanning of qualitative and quantitative ions (GB 23200.8-2016).

To screen differential accumulated metabolites (DAMs) among *C. kwangsiensis* comparison groups, an orthogonal partial least squares discriminant analysis (OPLS-DA) model was first constructed, and the Variable Importance in Projection (VIP) score was further calculated (with VIP>1 as the threshold) for initial identification of target metabolites. DAMs were identified based on their log2FC values (FC≥1) and VIP scores (VIP ≥1). Metabolite profiling data analysis was performed using MetaboAnalyst software. Principal Component Analysis (PCA) was utilized to assess the variation among different sample groups, meanwhile, the KEGG (Kyoto Encyclopedia of Genes and Genomes) pathway database was utilized to conduct functional annotation of DAMs.

### 2.3 RNA extraction and library sequencing

RNA was extracted from three tissues (leaves, rhizomes, and tubers) of *C. kwangsiensis* using the EASYspin Plus Complex Plant RNA Kit (Aidlab, Beijng, China). Afterward, 3 μg of RNA was used for transcriptome library preparation, with the library undergoing quality assessment via the Agilent 2100 Bioanalyzer (Agilent, Santa Clara, CA, USA). Upon passing the assessment, sequencing was performed on the Illumina HiSeq™ 2500 platform (Illumina, San Diego, CA, USA), producing 150 bp paired-end reads. After conducting quality control on the RNA sequencing data, the alignment of transcripts was carried out through Trinity (Haas et al., 2013). The quantification of mRNA expression was then determined in transcripts per million (TPM) using StringTie (v2.2.3) (Pertea et al., 2015). The quantification of mRNA expression was then determined in transcripts per million (TPM) using StringTie (v2.2.3) (Chen et al., 2021). Gene/transcript expression levels were quantified using FPKM(Fragments Per Kilobase Million) and the statistical package EdgeR in R software (Robinson et al., 2010). The DESeq2 software was utilized to detect differentially expressed genes (DEGs), applying criteria of |log2 (FC)| ≥1 and *p*-value ≤ 0.05 for selection.

### 2.4 Identification of *CkTPS* gene family and sequence analysis

The models for terpene synthase (PF01397) and terpene synthase C (PF03936) were obtained from the Pfam online repository (http://pfam.xfam.org/), each of which encompasses the *TPS* domain. The *TPS* gene family in the *C. kwangsiensis* within the annotated protein files from transcriptome was identified using the Simple HMM search in TBtools software (Chen et al., 2023). Gene sequences with an E-value < 1 × 10^−5^ were retained, while those sequences carrying both the PF01397 and PF03936 domains were further filtered through the Conserved Domain Database (CDD) and Pfam analysis (Mistry et al., 2021; Wang et al., 2023).

The gene sequences of *Arabidopsis thaliana* and *Solanum lycopersicum* were generated from the NCBI database. MEGAX 11 software was employed to align the *TPS* gene family protein sequences from *C. kwangsiensis*, *A. thaliana*, and *S. lycopersicum*, and ClustalW 2 software was used for conducting multiple sequence alignment analysis. The phylogenetic tree was constructed via the neighbor-joining (NJ) method and visualized using the iTOL. The protein properties of *CkTPS* genes, including size, relative molecular weight, theoretical isoelectric point (pI), and instability index, were analyzed using the ProtParam tool on the ExPASy platform (https://web.expasy.org/protparam/). Subcellular localization was predicted using the Cell-PLoc plant multi-localization predictor (http://www.csbio.sjtu.edu.cn/bioinf/plant-multi/).

### 2.5 Quantitative real-time polymerase chain reaction analysis

A total of 1 μg RNA underwent reverse transcription to cDNA for subsequent analysis using quantitative real-time PCR (qRT-PCR) with the Maxima H Minus First Strand cDNA Synthesis Kit by Thermo Scientific, based in Waltham, MA, USA. The specific experimental procedure conditions are as follows: pre-denaturation at 95°C for 10 min, followed by 40 amplification cycles (95°C for 10 s, 60°Cfor 10 s, 72°C for 20 s each), and a subsequent melt curve analysis (denaturation at 95°C for 5 s, annealing at 65°C for 1 min). The 2^-ΔΔCt^ method was utilized to analyze the relative gene expression, with the *Actin* gene serving as the internal reference gene. The study included three biological replicates, and the primers used are detailed in **Supplementary Table S2**.

### 2.6 Cloning of the candidate *CkTPS* gene

Based on the transcriptome sequences, PCR primers were designed (**Supplementary Table S3**). The PCR products were added to the pTopo-Blunt Cloning Vector, and subsequently transformed into DH5α *E.coli* competent cells using the heat shock method. The positive clones that were identified were dispatched to Tsingke Biotech in Beijing, China for further processing. Following this, the appropriate colonies were transferred and left to incubate overnight. Subsequent analyses were carried out on the extracted plasmids using a TianGen Plasmid Mini Kit.

### 2.7 Heterologous expression of in the candidate *CkTPS10* gene and activity assay

In the context of *in vivo* enzyme assays, INVSc1 was utilized as the corresponding host strain. The candidate *CkTPS10* gene was amplified by the pESC-Ura-*CkTPS10*-F and pESC-Ura-*CkTPS10*-R primers (**Supplementary Table S2**). The yeast was transfected with vector pESC-Ura containing *CkTPS10* and with an empty vector pESC-URA using electroporation. The transformed yeast strains were subsequently confirmed through polymerase chain reaction (PCR) analysis to validate the presence of the recombinant DNA. After transforming the yeast, the recombinant strains were streaked on SD with agar plates and incubate at 30 °C for 2 days. Then, the yeast colonies from the plates were washed using the liquid medium without uracil (SD-Ura) containing 2% lactose, and measured the OD value and dilute it to an OD_600_ of 1.0. Then, though inoculating 50 uL of the above yeast culture into 10 mL of YPD liquid medium containing 2% lactose, the yeast cells were promoted the expression of goal proteins. After continuous cultivation and incubation at 30°C with shaking at 200 rpm for 24 hours, 1 μL of the substrate farnesyl pyrophosphate (FPP) and 2 mL of dodecane were added to the culture. The mixture was further incubated at 30°C with shaking at 200 rpm for 48 hours. Following incubation, the organic phase was separated via centrifugation at 5000 rpm for 10 min at 4 °C. The dodecane layer was collected, filtered through a 0.22-μm nylon membrane, and analyzed by GC-MS according to the method described by Liu et al., 2012.

### 2.8 Statistical analysis

All experimental results are expressed as mean ± standard deviation (SD), with each sample subjected to three biological replicates. Statistical analysis was performed using GraphPad Prism 9.5 software, and the significance of differences between groups was determined by Student’s t-test, where “ns” *p* > 0.05, *p < 0.05, **p < 0.01, ***p < 0.001.

## 3 Result

### 3.1 Metabolic profiling of different tissues in *Curcuma kwangsiensis*

**Figure 1:**
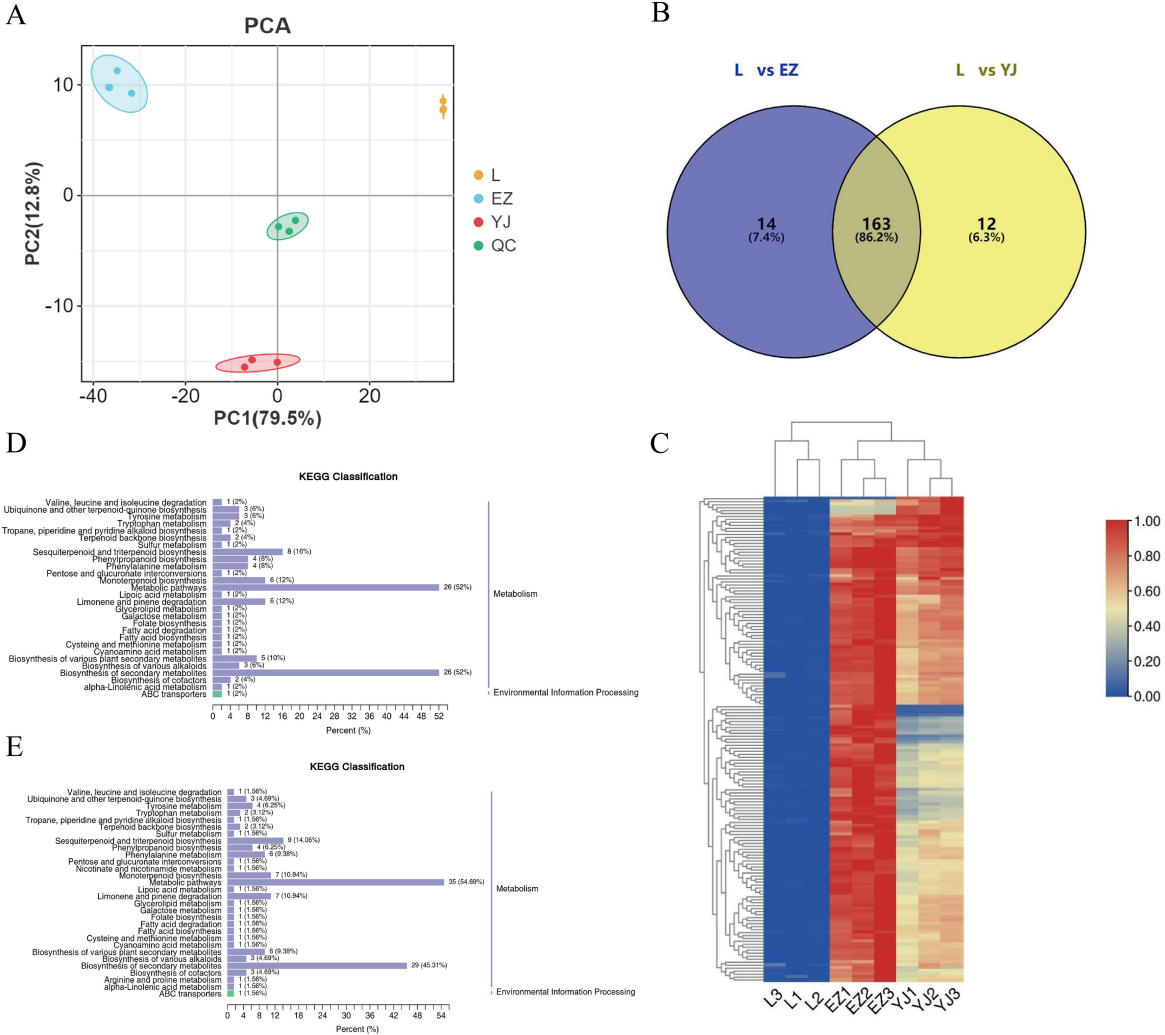
Analysis of differential metabolites in *C.kwangsiensis*. **(A)** PCA scores of the mass spectrometry data of each group of *C.kwangsiensis* and the quality control samples, L: leaf; EZ: rhizome; YJ: tuber. **(B)** Distribution of terpenoid differential metabolites in rhizomes and tubers of *C.kwangsiensis*. **(C)** Cluster analysis of differential metabolites of *C.kwangsiensis.***(D)** KEGG classification of differential metabolites in L vs EZ group of *C.kwangsiensis*. **(E)** KEGG classification of differential metabolites in L vs YJ group of *C.kwangsiensis*.

To explore the differential metabolite changes of the medicinal parts of *C.kwangsiensis*, we collected the fresh leaves, rhizomes and tubers for metabolomic analysis (**Supplementary Figure S1**). Among them, with leaves as the control, they were divided into two groups for comparison, namely leaves vs rhizomes (L vs EZ) and leaves vs tubers (L vs YJ). A comprehensive assessment of volatile metabolite alterations in various tissues of *C.kwangsiensis* was achieved through metabolomic profiling analysis conducted with an HS-SPME-GC-MS system. As shown in the Principal Component Analysis (PCA) score plot, the three tissue types of *C. kwangsiensis* exhibited distinct separation from one another. This observation suggests that there are significant variations in the distribution patterns of volatile metabolites across these tissues (**Figure 1A**). Based on the OPLS-DA model, a significant number of differential metabolites were identified in leaves, rhizome, and tubers. Specifically, 773 differential metabolites were detected in L vs EZ group, among which 177 terpenoids were upregulated. A total of 718 differential metabolites were identified in L vs YJ, with 175 terpenoids upregulated in tubers. Comparative analysis revealed 163 shared upregulated terpenoid metabolites between the two groups (**Figure 1B**, **Supplementary Table S4**). Meanwhile, the Heatmap demonstrated high repeatability among the samples from different tissues, supported the PCA analyses results. Overall, the enrichment levels of metabolites in tubers and rhizome tissues were significantly higher than those in leaves (**Figure 1C**).

Based on KEGG pathway categories, the significantly different metabolites were annotated and classified. The results showed that the two groups of differential metabolites were primarily involved in two major pathways: Metabolism and Environmental Information Processing, with metabolic pathways accounting for the largest proportion. The significantly enriched pathways for differential metabolites included Metabolic pathways, Biosynthesis of secondary metabolites, Sesquiterpenoid and triterpenoid biosynthesis, Monoterpenoid biosynthesis, Limonene and pinene degradation, and Biosynthesis of plant secondary metabolites. Notably, a substantial number of metabolites were also enriched in Terpenoid backbone biosynthesis and Sesquiterpenoid and triterpenoid biosynthesis (**Figure 1D, E**). Further characterization of differential metabolites enriched in Sesquiterpenoid and triterpenoid biosynthesis and Terpenoid backbone biosynthesis pathways showed that 9 metabolites were identified, including β-Selinene, cis-trans-Farnesol, Nerolidol, δ-Cadinene, α-Farnesene, trans-γ-Bisabolene, trans-Farnesol, trans,trans-Farnesal, and (-)-Geosmin. Notably, eight of these differential metabolites were all upregulated and belonged to terpenoids, whereas one belonged to aromatic hydrocarbons and was downregulated (**Supplementary Table S5**).

### 3.2 Transcriptomic analysis of different tissues *Curcuma kwangsiensis*

For clarifying the key enzyme genes that contribute to the sesquiterpene biosynthesis process, sequencing was performed on total RNA isolated from different organ tissues. The sequencing reads were processed through image recognition, contamination removal, adapter trimming, and low-quality sequence filtering. Subsequently, the statistical results showed that the Q30 and Q20 values of Clean Reads exceeded 87.5% and 95.5%, respectively, with GC content ranging from 43.8% to 49.4% (**Supplementary Table S6**), indicating that the transcriptome sequencing data of *C. kwangsiensis* are of high quality and reliable.

**Figure 2:**
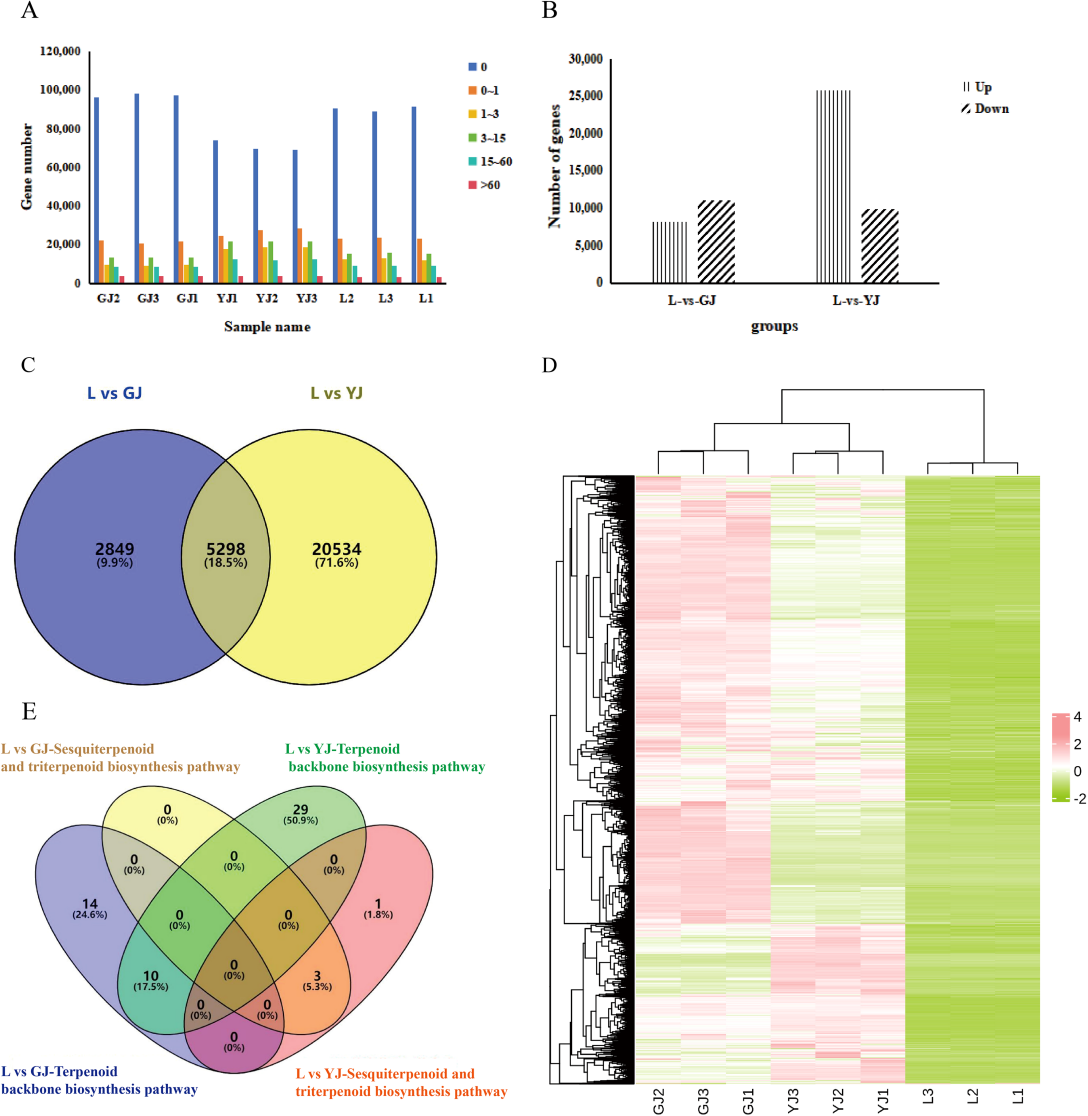
Comprehensive analysis of DEGs in *C.kwangsiensis*. L: leaf; GJ: rhizome; YJ: tuber. **(A)** Distribution of gene expression. **(B)** Comparison of the number of up-regulated and down-regulated differential genes in different tissues of *C.kwangsiensis*. **(C)** Venn diagram of DEGs. The numbers in each region represent the count of DEGs, and the percentages in parentheses indicate the proportion of DEGs in the corresponding comparison. The overlapping region expressed in both L vs GJ and L vs YJ comparisons, while 2849 DEGs are specific to L vs GJ, and 20534 DEGs are specific to L vs YJ. **(D)** Heat map of differential gene expression clustering, green: low expression; red: high expression. **(E)** Distribution of differential gene enrichment on terpene biosynthesis-related pathways.

Due to lack suitable reference genome for gene function annotation of *C. kwangsiensis*, a total of 153,831 genes were successfully annotated through transcriptome database alignment (**Supplementary Table S1**). In all samples, most of genes remained unexpressed, and the fewest genes exhibited higher expression level (FPKM > 60). Notably, compared to the other two tissues, the tuber (YJ) displayed a relatively higher abundance of expressed genes (**Figure 2A**), which implied the gene reprogramming in the tubers of *C. kwangsiensis* is more complex, resulting in greater changes in its transcriptional level. The multiple differentially expressed genes (DEGs) were detected in the comparisons of leaves vs rhizome (L vs EZ) and leaves vs tuber (L vs YJ). Specifically, in the comparison between the two groups L and YJ, the highest number of DEGs was identified, totaling 35,716, with 25,832 showing significant upregulation and 9,884 displaying downregulation. Moreover, a total of 19,265 DEGs were identified between leaves and rhizomes, including 8,147 upregulated and 11,118 downregulated genes (**Figure 2B**). A Venn diagram of upregulated DEGs from the two groups revealed 5,289 genes that were simultaneously upregulated in both comparisons (**Figure 2C**).

Cluster heatmap results showed that the majority of DEGs exhibited the highest expression levels in root and stem tissues, followed by tuber tissues, whereas nearly all DEGs displayed low expression in leaves (**Figure 2D**). In order to identify the genes that control the sesquiterpene biosynthetic pathway in *C. kwangsiensis*, numerous upregulated DEGs in the leaf vs. rhizome and leaf vs. tuber comparison groups were further identified. KEGG analysis indicated that the two groups of upregulated DEGs were found to be enriched in terpenoid synthesis-related pathways. Total 53 differentially expressed genes were enriched in the terpenoid backbone biosynthesis pathway, and 4 differentially expressed genes were enriched in the sesquiterpenoid and triterpenoid biosynthesis pathways. Notably, of the 57 key DEGs, only one gene, TRINITY_DN14871_c0_g3, encoded terpenoid synthases (*TPS*) in *C. kwangsiensis* (**Figure 2E**, **Supplementary Table S7**).

### 3.3 Correlation analysis with DAMs and DEGs

**Figure 3:**
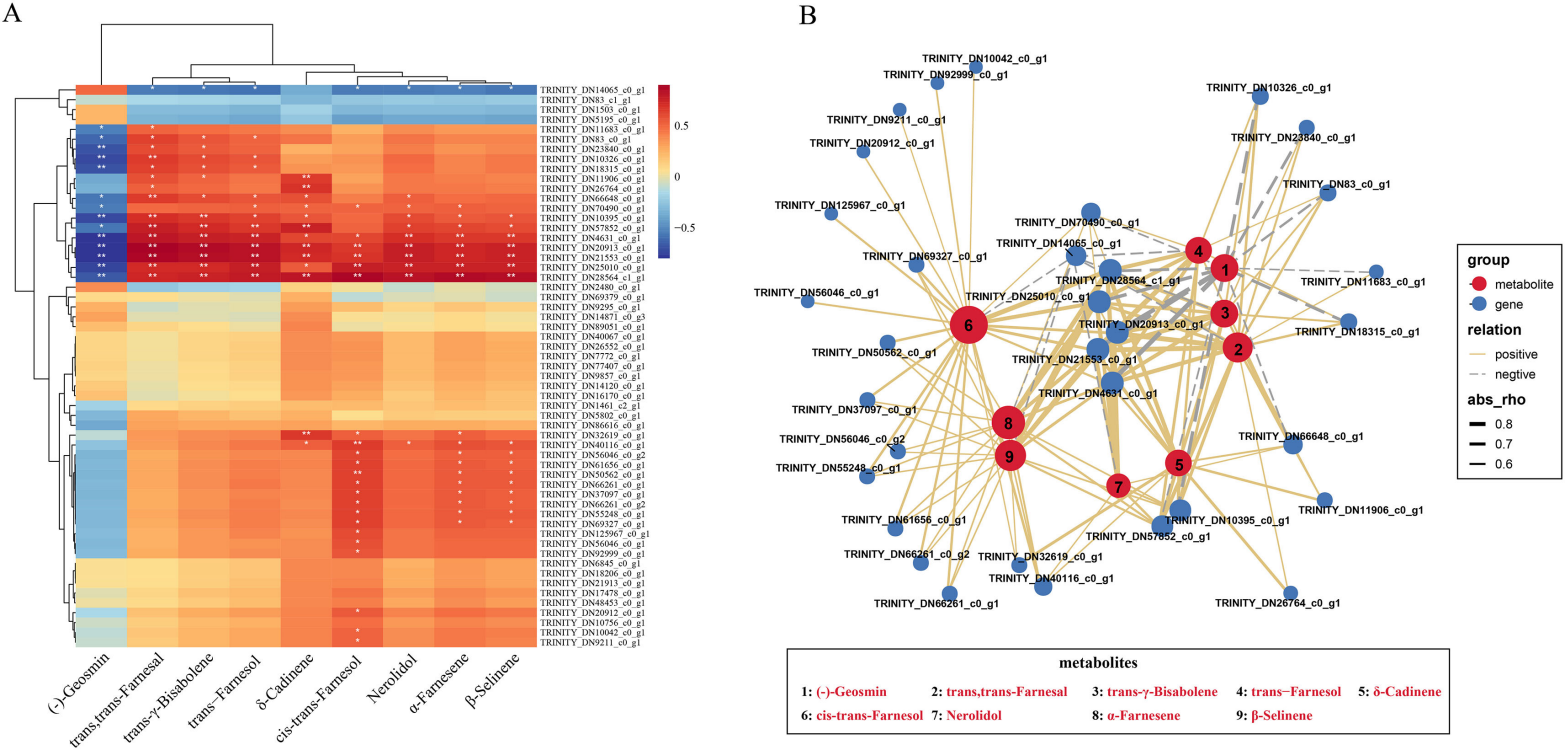
Correlation analysis of differential expressed genes and differential accumulation metabolites in the rhizomes and tubers tissues of *C. kwangsiensis*. **(A)** Heatmap of spearman correlations between 57 sesquiterpene synthesis-associated upregulated DEGs and 9 DAMs in Rhizome/Tuber Tissues of *C. kwangsiensis* (Significant correlations at *p* < 0.05 are marked with white stars) **(B)** Correlation network of 33 upregulated DEGs involved in sesquiterpene synthesis and 9 DAMs with significant associations (|r| > 0.5, *p* < 0.05) in *C. kwangsiensis* rhizomes and tubers. Red dots indicate DAMs, blue dots indicate DEGs; Positive correlations are indicated by solid lines, and negative correlations by dotted lines—with the line thickness reflecting the absolute magnitude of the correlation.

Based on the above metabolomic and transcriptomic data of *C. kwangsiensis* throughout leave, rhizomes and tubers, the association analysis of these two omics data was carried out to clarify the correlation between DAMs and DEGs, aiming to further screen key genes of sesquiterpenoid biosynthetic pathways. Through Pearson analysis of nine key DAMs and fifty-seven up-regulated DEGs related to sesquiterpenoid synthesis in tuber and rhizome tissues, the findings demonstrated a clear association (|r| > 0.5, *p* < 0.05) between the expression patterns of 33 out of the 57 DEGs and the patterns of accumulation of particular metabolites that exhibited differential levels. (**Figure 3A**). Subsequently, a network integrating these interconnected genes and corresponding metabolites was then built. The findings demonstrated that eight sesquiterpenoids of differential metabolites exhibited positive correlations with 32 candidate differential expressed genes, except for one gene TRINITY_DN14065_c0_g1 (encoded 4-diphosphocytidyl-2-C-methyl-D-erythritol kinase, CMK), while geosmin (aromatic) showed negative correlations with multiple differential genes (**Figure 3B**). It is worth noting that no genes encoded terpenoid synthases (*TPS*) for sesquiterpene synthesis were found among these 33 key DEGs of *C.kwangsiensis*, suggesting that the *CkTPS* may be involved in the synthesis of other active sesquiterpene components.

### 3.4 Identification of *CkTPS* gene family

To further mine more *TPS* genes for sesquiterpene synthesis in *C. kwangsiensis*, we conducted *TPS* family gene identification based on transcripts database. In this study, a total of 24 *TPS* members, designated as *CkTPS1* to *CkTPS24*, were identified via HMMER and BLASTP searches. The lengths of *CkTPS*-encoded protein sequences differed, where the minimum length was 251 amino acids (*CkTPS6*) and the maximum reached 1301 amino acids (*CkTPS12*). The theoretical isoelectric points (pI) spanned 4.80 to 8.67 and the instability indices ranged from 32.22 to 56.90. Subcellular localization prediction results indicated that 12 out of the total *CkTPS* proteins were predicted to be localized in the chloroplast, and the rest were likely present in the cytoplasmic compartment, and two proteins were predicted to undergo extracellular secretion. Further information of the 24 *CkTPS* genes is available in **Supplementary Table S9**.

**Figure 4:**
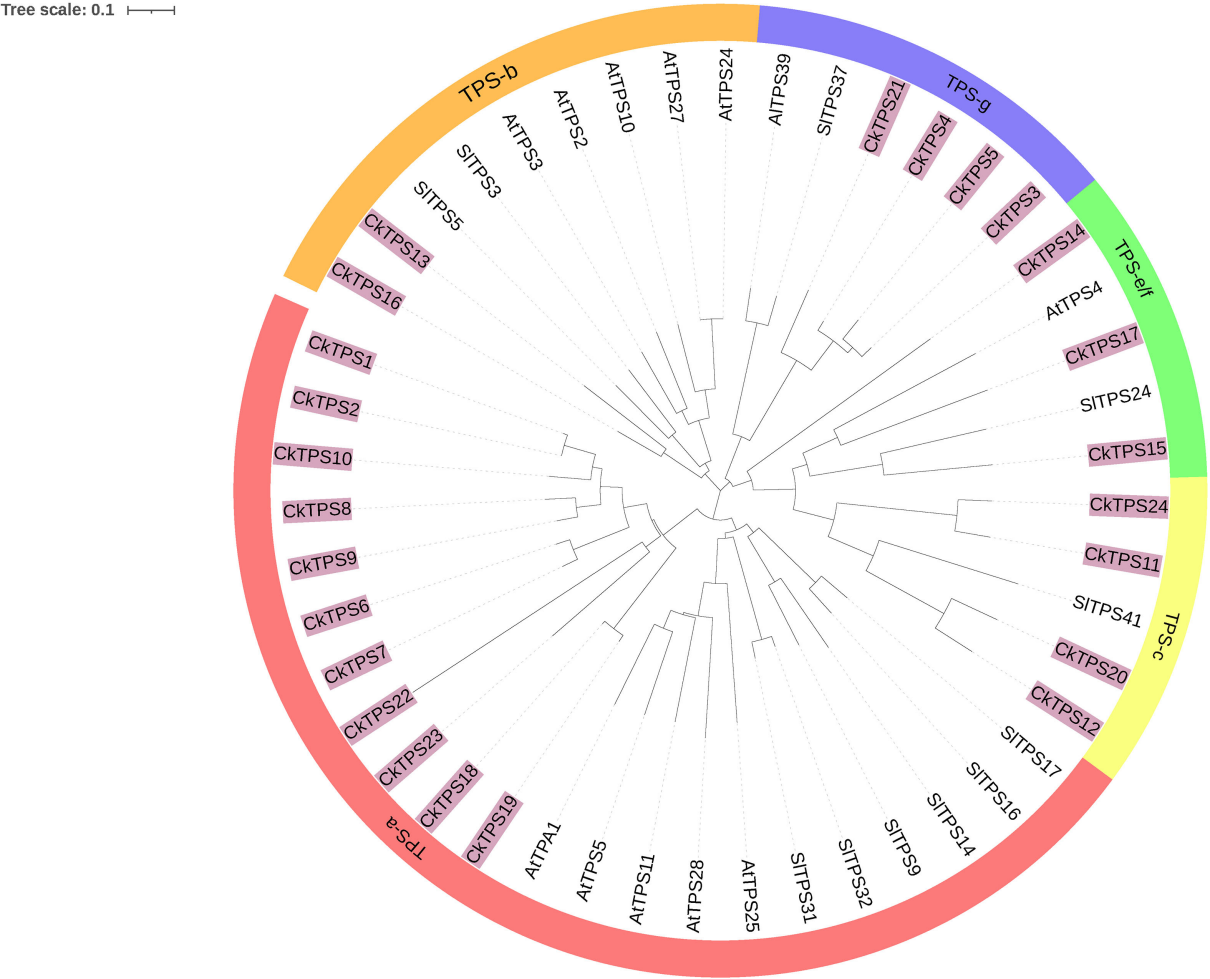
Phylogenetic tree of *TPS* gene family in C.kwangsiensis, Arabidopsis thaliana, and Lycopersicon esculentum.

To enhance comprehension of the evolutionary dynamics of *CkTPS* genes, a phylogenetic analysis was performed using 24 *CkTPS* proteins and compared with known *TPS* proteins from *Arabidopsis thaliana* (Parker et al., 2014) and *Solanum lycopersicum* (Falara et al., 2011). The 24 *CkTPS* genes in *Arabidopsis thaliana* were classified into five evolutionary clades based on the classification method for *TPS* proteins. (**Figure 4**). The *TPS-a* subfamily harbored the largest number of family members, including 11 *CkTPS* genes (*CkTPS1、CkTPS2、CkTPS6 ~ CkTPS10、CkTPS18、CkTPS19、CkTPS22* and *CkTPS23*), while the *TPS-b* subfamily contained only two members (*CkTPS13* and *CkTPS16*). Moreover, the *TPS-e/f* subfamily comprised three genes (*CkTPS14*、*CkTPS15* and *CkTPS17*), and both *TPS-c* and *TPS-g* subfamilies each consisted of four *CkTPS* genes.

**Figure 5:**
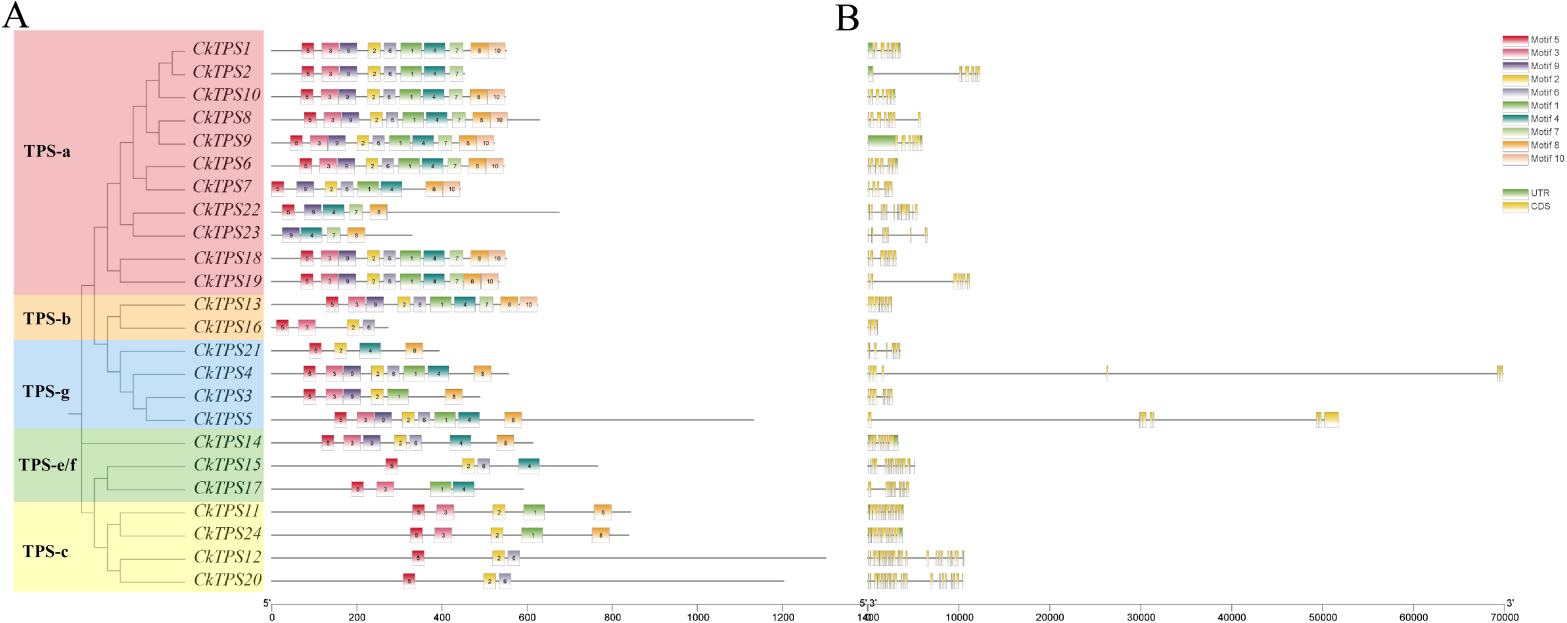
Conserved motif and gene structures of *TPS* genes in *C.kwangsiensis***(A)** conserved motif; **(B)** gene structures.

As illustrated in **Figure 5A**, *CkTPS* proteins that clustered in the same phylogenetic group displayed similar motif compositions as well as consistent configurations. Motif analysis showed that *TPS-a* subfamily members harbored a higher number of conserved motifs, while *TPS-e/f* and *TPS-c* subfamilies had fewer conserved motifs. Interestingly, the first conserved motif of most *CkTPS* genes was consistently identified as Motif 5. Except for the *TPS-a* subfamily, other *TPS* subfamily members mostly lacked Motif 7 and Motif 10. Moreover, Gene structure analysis revealed that *TPS-e/f* and *TPS-c* subfamily members contained more exons (**Figure 5B**).

### 3.5 Validating transcriptome sequencing data using real-time quantitative PCR technology

**Figure 6:**
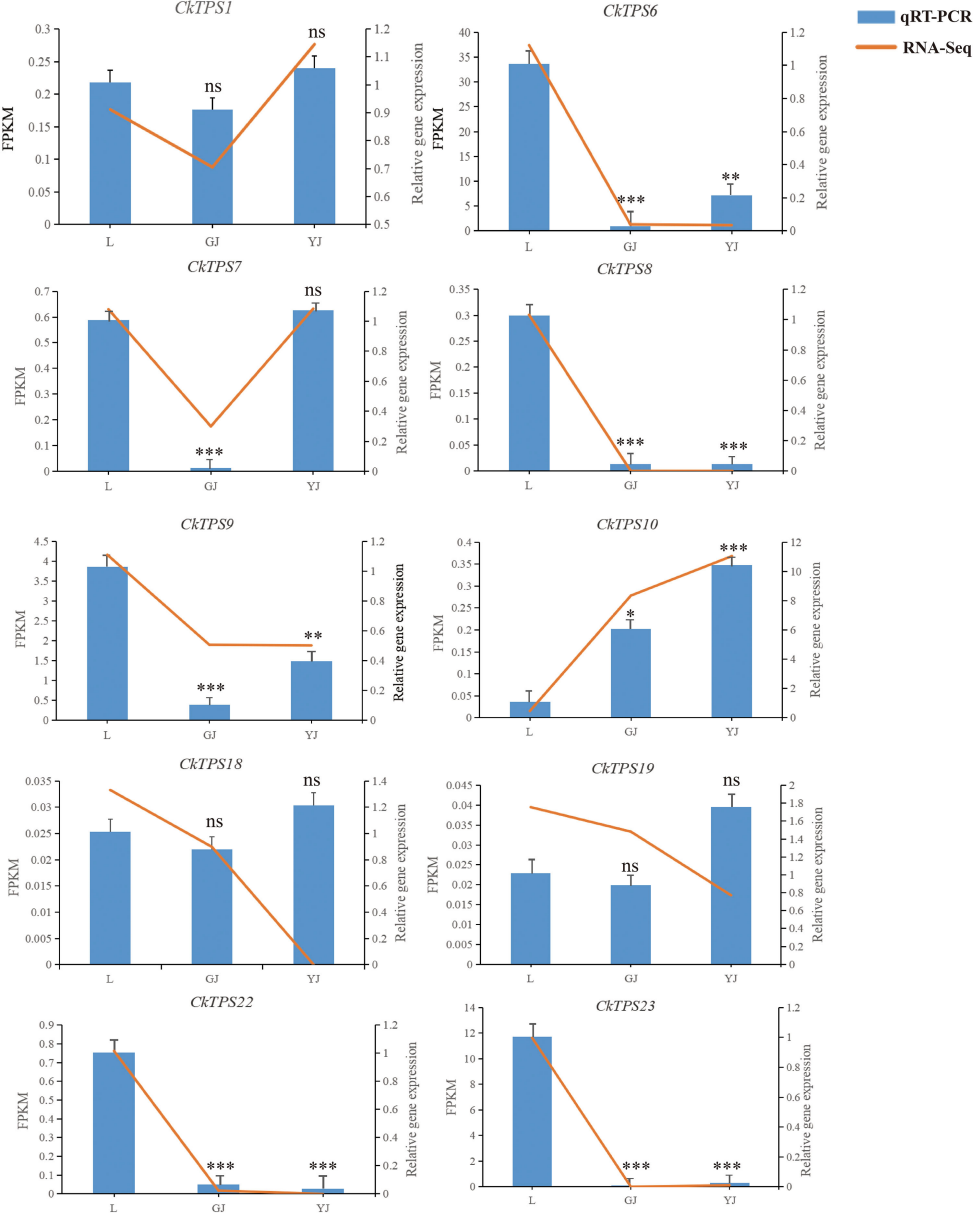
Comparison of RT-qPCR and transcriptome result of *TPS-a* subfamily gene expression. The horizontal axis refers to different tissue parts. L: leaf; GJ: rhizome; YJ: tuber. The left vertical axis represents gene FPKM in RNA-seq data, and the right vertical axis represents the relative expression level of *TPS* genes by RT-qPCR. “ns”indicated p>0.05, * indicates significant difference, *p < 0.05, **p < 0.01, ***p < 0.001.

To ascertain the dependability of the transcriptomic data and elucidate the expression patterns of *TPS* gene family members in diverse tissues of *C. kwangsiensis*, RT-qPCR analysis was performed on 10 selected genes from the *TPS-a* subfamily in all experimental specimens. As shown in **Figure 6**, the most *TPS-a* subfamily members exhibit higher expression in leaves or tubers. *CkTPS6、CkTPS8*、*CkTPS9、CkTPS22* and *CkTPS23* displayed the higher expression in leaves, whereas *CkTPS10* showed tuberous root-specific expression. Remarkably, *CkTPS10* achieved high expressions in both rhizome and tuberous roots compared with leave. The above results are consistent with the transcriptome data, reflecting the authenticity and reliability of the transcriptome data.

### 3.6 Functional identification of *CkTPS10* using the yeast system

**Figure 7:**
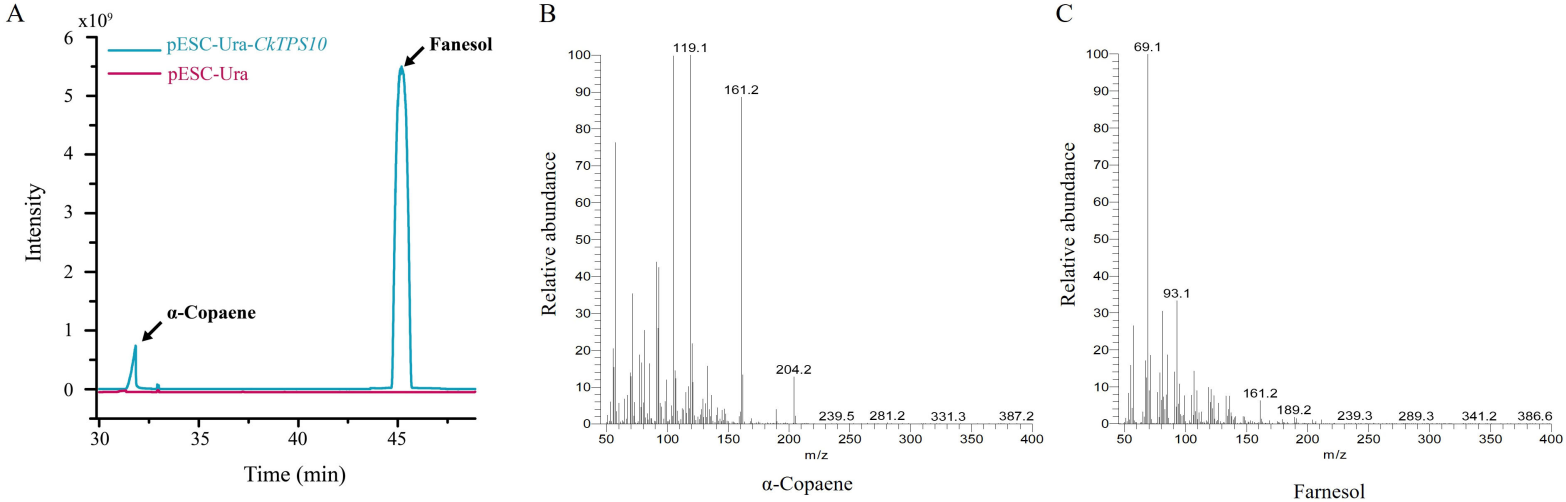
*In vivo* based functional validation of *CkTPS10*. **(A)** GC analysis of *in vivo* assays of pESC-Ura-*CkTPS10* and empty vector with FPP substrate. pESC-Ura-*CkTPS10*, yeast harboring pESC-Ura-*CkTPS10*; pESC-Ura, yeast carrying empty vector; **(B)** Mass spectral fragmentation of detected product: α-copaene. **(C)** Mass spectral fragmentation of detected product: Farnesol.

It is well known that there is a close association between the *TPS-a* subfamily and sesquiterpene biosynthesis. Based on the above gene expression of *TPS-a* subfamily (**Figure 6**), *CkTPS10* was selected as the candidate gene for further gene cloning and heterologous expression. To assess the enzymatic activity of *CkTPS10*, we introduced and expressed the cDNA corresponding to this enzyme in yeast. After 48 h of galactose-mediated induction of gene overexpression, the volatile compound profiles of the recombinant pESC-Ura-*CkTPS10* protein were characterized and identified via GC-MS to clarify its chemical composition. The yeast-derived compounds were matched against the NIST database (The NIST version 2.2, 2020). for identification. This study indicated that strains with the *CkTPS10* variant converted FPP into α-copaene and farnesol, and these two compounds were determined to be the major final products of this reaction. This finding confirms that the *CkTPS10* enzyme has functional sesquiterpenoid synthase activity. (**Figure 7**, **Supplementary Table S10**). The results indicated that *CkTPS10* is capable of utilizing FPP as a base and facilitating the synthesis of a blend of sesquiterpenoids.

## 4 Discussion

### 4.1 Metabolomics analysis of *Curcuma kwangsiensis*

As an aromatic zingiaceae plant, *C.kwangsiensis* contains rich volatile oil, in which there are a large amount of sesquiterpene components, most of which are the unique pharmacodynamic substances of *C.kwangsiensis*, and these components have obvious therapeutic effects in anti-tumor, cardiovascular and liver diseases (Gao et al., 2021; Wang et al., 2018; Zeng et al., 2012). Therefore, research on sesquiterpenoids from this medical plant has been a hot topic in recent years. Metabolomics is a discipline that studies the metabolome - the collection of all metabolites within a cell at a certain moment. This research not only sheds light on the crucial signaling molecules that govern the inner transformations in plants, but also paves the way for delving into the pivotal genes involved in sesquiterpene synthesis through comprehensive multi-omics approaches (Gao et al., 2022). However, there are few metabolomic studies on sesquiterpenes of *C.kwangsiensis*, which limits more systematic and complete studies on the sesquiterpenes synthesis of *C.kwangsiensis* and cannot provide relevant support for development of new drugs.

In this report, a comprehensive characterization of metabolites in three tissues of *C. kwangsiensis* was conducted via metabolomics analysis. There were 177 and 175 terpenoid differential metabolites upregulated in the rhizome and tuber compared to the leaf, respectively. Among these, 163 upregulated terpenoid differential metabolites were shared between the two groups, including the pharmacologically active compounds of *C. kwangsiensis* such as curcumol, curgerenone, and β-elemene, which aligns with the traditional Chinese medicine practice of using the rhizome and tuber of *C. kwangsiensis* for medicinal purposes. Recently, Zhou et al. (2025) have found 197 DAMs, encompassing sesquiterpenoid compounds (germacrone, curzerenone, β-elemen, curcumenol) that are accumulated during the expansion stage of tuber development in *C. kwangsiensis*. Although the findings were partially similar to the analysis results of differential metabolite in our study, we have more clearly identified the differential metabolites between roots and tubers by multi-tissue comparison. Through KEGG classification, a high abundance of metabolites was further identified in the Sesquiterpenoid and triterpenoid biosynthesis pathway, with nine metabolites annotated—eight of which were sesquiterpenoids in rhizome and tuber, proposing that sesquiterpene derivatives are prone to accumulation in the roots of *C. kwangsiensis* instead of its aerial parts. This phenomenon has also been found in other medicinal plants, such as Atractylodes and Bupleurum (Qu et al., 2022; Ruan et al., 2021). However, more studies could focus on these key differential metabolites to further explore the different molecular mechanisms of sesquiterpenoid biosynthesis between the rhizomes and tubers in *C. kwangsiensis*.

### 4.2 Transcriptome analysis of *Curcuma kwangsiensis*

Due to advancements in high-throughput sequencing technology and the decrease in sequencing expenses, RNA-seq technology has found extensive utilization in the investigation of traditional Chinese medicinal substances. Through profiling of transcriptome, Xu et al. (2025) dissected multiple enzymes and Unigenes involved in polyphyllin biosynthesis among the three *Paris* species, genes encoding enzymes such as 2,3-oxidosqualene cyclases (OSCs), cytochrome P450s (CYPs), and UDP glycosyltransferases (UGTs). In *Atractylodes lancea*, A previous study successfully pinpointed 80 genes linked to the biosynthesis pathway involved in sesquiterpene production. Further analysis of these genes revealed that 13 of these genes, categorized as terpene synthase(*TPSs*)-encoding genes, play a direct role in producing the sesquiterpene compounds that accumulate in the rhizomes of the target plant (Zhang et al., 2025). Thus, multi-omics analyses based on transcriptomics and metabolomics have increasingly become one of the effective strategies for investigating distinct metabolic pathways and their corresponding regulatory genes.

In our study, RNA-seq technology was employed to construct transcriptome database among three tissues of *C.kwangsiensis*, leading to offer abundant sequence information for investigating the biosynthetic pathways and mechanisms of diverse terpenenoids. In secondary metabolism, 57 up-regulated DEGs were collectively enriched in Terpenoid backbone biosynthesis and Sesquiterpenoid and triterpenoid biosynthesis pathways in rhizome and tuber. Specifically, the candidate gene *CkTPS10* was included, inferring these candidate genes may be the molecular keys for the formation of important active components of *C. kwangsiensis*. Through integrative analysis of metabolomics and transcriptomics, it was found that the differential metabolites were positively correlated with most of the differential genes, while only (-)-Geosmin was negatively correlated with the differential genes. This is related to the fact that (-)-Geosmin is a non-terpene differential metabolite and shows a downregulated expression trend, further confirming the reliability of the correlation network diagram. However, we found that the correlation between the *CkTPS10* gene and eight sesquiterpene metabolites was not high (*p* < 0.5), suggesting that this candidate gene might be involved in the synthesis of other active components, which was indeed confirmed by subsequent functional characterization.

### 4.3 Identification of *TPS* gene family of *Curcuma kwangsiensis*

Terpenoid compounds serve as the active constituents in numerous plants, and *TPS* plays an important role in regulating the biosynthesis of various terpenoids (Bergman and Dudareva, 2024). The *TPS* gene family is a medium-sized family with highly diverse sequences and functions, containing approximately 20 to 180 genes in the plant genome (Shang et al., 2020; Yu et al., 2021; Jiang et al., 2024). In recent years, there has been a growing trend in the identification and exploration of *TPS* gene families across various medicinal plant species. There were 48 *EpTPS* family members in *Entada phaseoloides*, which were classified into six subgroups (Lin et al., 2022). Based on integrated genomic and transcriptomic data of *Dendrobium officinale* (Yu et al., 2020) identified 34 *TPS* genes, and the expression of *DoTPS* genes was chiefly observed in flowers, with roots and stems showing lower levels of expression. A comprehensive comparative genomics analysis revealed the presence of 75 putative *WlTPS* in *W. longiligularis*, with *WlTPS*24/26/28 specifically linked to borneol diphosphate synthase (BPP) functionality (Yang et al., 2023). However, the genomic information of *Curcuma*, which also belongs to the Zingiaceae family, has not been documented yet, thus impeding investigations into terpene biosynthesis mechanisms and the improvement of Curcuma varieties through selective breeding.

The increasing exploration of *TPS* have been achieved to provide a comprehensive insight on the biosynthesis and regulatory mechanisms of terpenoids in plants. To date, all the *CkTPS* genes can be classified into five subfamilies (*TPS*-a to *TPS*-g) in *C. kwangsiensis*, and the *TPS*-a subfamily comprises the largest number of *CkTPS* members, similar to *TPS* subfamily distributions in most aromatic plants (Zhou et al., 2022; Xi et al., 2025). Notably, it is reported that *TPS*-a is closely associated with sesquiterpene biosynthesis, which aligns with sesquiterpenoids being the primary components of *C. kwangsiensis*. In addition, the same subfamily members share similar motif compositions and arrangements, indicating potential functional similarities within subfamilies. Except *TPS*-a, most subfamily members lack Motif 7 and Motif 10, implying that these two conserved motifs are speculated to be associated with sesquiterpene synthase activity.

### 4.4 Functional characterization of *CkTPS*

Given the role of *TPS* genes in shaping terpene structural diversity, an expanding number of *TPS* gene functions are being characterized. Protein function determination commonly employs two approaches: *in vivo* or *in vitro* validation. *In vivo* validation predominantly utilizes eukaryotic organisms like plants and yeast, whereas *in vitro* validation entails the isolation and purification of recombinant proteins through prokaryotic expression systems. Through yeast heterologous expression and *in vitro* enzyme assays, *XsTPS1* catalyzes the formation of multiple compounds, predominantly germacrene D, whereas β-caryophyllene synthesis is catalyzed by *XsTPS3* (Li et al., 2016). Fujita et al. (2017) demonstrated *ZpTPSs* primarily catalyze the biosynthesis of β-phellandrene through heterologous expression in *Escherichia coli* and *Agrobacterium*-mediated transient expression in *Nicotiana benthamiana*. To date, investigations on turmeric have solely employed prokaryotic expression systems and tobacco heterologous expression for validating the role of *TPS* in *wenyujin* (Jiang et al., 2021), with yeast system technology remaining unutilized.

Given the limited number of *TPS* genes with validated functions in *C.kwangsiensis*, there is a critical need for systematic exploration and functional validation of these genes. Based on the prior identification and analysis of the *CkTPS* gene family, we screened one *TPS* gene *CkTPS10* for preliminary functional characterization. Interestingly, this candidate gene did indeed appear in the candidate gene cluster of differentially expressed genes (**Figure 2**, **Supplementary Table S7**). However, its protein did not catalyze the generation of those eight candidate differentially enriched metabolites related-terpene synthesis via the two-omics correlational analysis (**Figure 3**, **Supplementary Table S5**). The GC-MS results also confirmed this point, employed a yeast heterologous expression system to validate the functionality of candidate *CkTPS* gene. The result demonstrated the catalytic products of *CkTPS10* indeed contained only two sesquiterpenoids, α-copaene and farnesol, however, which correspond to total key differential metabolites identified in *C.kwangsiensis* (**Supplementary Table S1**1). In conclusion, the findings corroborated that the screened *CkTPS10* gene played a crucial role in sesquiterpenoid biosynthesis and its protein exhibit the function of catalyzing sesquiterpene formation in *C.kwangsiensis*.

## 5 Conclusion

In this study, through constructing metabolomic and transcriptomic databases of *C. kwangsiensis* (leaves, rhizomes, and tubers) and performing integrated analysis, a close correlation was confirmed between differentially expressed genes and variations in differential metabolite contents in terpene synthesis pathway. The *TPS* gene family in *C. kwangsiensis* was systematically identified and analyzed, followed by functional characterization of the candidate *CkTPS* gene. HS-SPME combined with GS-MS results demonstrate that *CkTPS10* play a pivotal role in the biosynthesis of sesquiterpenoid compounds in this plant. This study is helpful to further understand the molecular mechanism of the synthesis and accumulation of volatile bioactive components in *C. kwangsiensis*. Moreover, it also can provide useful gene resources for metabolic engineering in *Saccharomyces cerevisiae* for future metabolic engineering.

## Data Availability

The transcriptomic raw data generated in this study have been deposited in the NCBI under the BioProject accession number PRJNA1347965. These data are publicly accessible through the NCBI SRA portal (https://www.ncbi.nlm.nih.gov/sra).

## References

[B1] Bergman M. E. Dudareva N. (2024). Plant specialized metabolism: Diversity of terpene synthases and their products. Curr. Opin. Plant Biol. 81, 102607. doi: 10.1016/j.pbi.2024.102607, PMID: 39053147

[B2] Chen F. Tholl D. Bohlmann J. Pichersky E. (2011). The family of terpene synthases in plants: a mid-size family of genes for specialized metabolism that is highly diversified throughout the kingdom. Plant J. 66, 212–229. doi: 10.1111/j.1365-313X.2011.04520.x, PMID: 21443633

[B3] Chen R. Wei Q. Liu Y. Wei X. Chen X. Yin X. . (2021). Transcriptome sequencing and functional characterization of new sesquiterpene synthases from Curcuma wenyujin. Arch. Biochem. Biophysics 709, 108986. doi: 10.1016/j.abb.2021.108986, PMID: 34252391

[B4] Chen C. Wu Y. Li J. Wang X. Zeng Z. Xu J. . (2023). TBtools-II: A "one for all, all for one" bioinformatics platform for biological big-data mining. Mol. Plant 16, 1733–1742. doi: 10.1016/j.molp.2023.09.010, PMID: 37740491

[B5] Das A. Begum K. Akhtar S. Ahmed R. Kulkarni R. Banu S. (2021). Genome-wide detection and classification of terpene synthase genes in Aquilaria agallochum. . Physiol. Mol. Biol. Plants 27, 1711–1729. doi: 10.1007/s12298-021-01040-z, PMID: 34539112 PMC8405786

[B6] Falara V. Akhtar T. A. Nguyen T. T. Spyropoulou E. A. Bleeker P. M. Schauvinhold I. . (2011). The tomato terpene synthase gene family. Plant Physiol. 157, 770–789. doi: 10.1104/pp.111.179648, PMID: 21813655 PMC3192577

[B7] Fu Z. Liu H. Kuang Y. Yang J. Luo M. Cao L. . (2025). β-elemene, a sesquiterpene constituent from Curcuma phaeocaulis inhibits the development of endometriosis by inducing ferroptosis via the MAPK and STAT3 signaling pathways. J. Ethnopharmacology 341, 119344. doi: 10.1016/j.jep.2025.119344, PMID: 39800242

[B8] Fujita Y. Koeduka T. Aida M. Suzuki H. Iijima Y. Matsui K. (2017). Biosynthesis of volatile terpenes that accumulate in the secretory cavities of young leaves of Japanese pepper (Zanthoxylum piperitum): Isolation and functional characterization of monoterpene and sesquiterpene synthase genes. Plant Biotechnol. 34, 17–28. doi: 10.5511/plantbiotechnology.16.1231a, PMID: 31275004 PMC6543703

[B9] Gao J. Li T. Jiao L. Jiang C. Chen S. Huang L. . (2022). Metabolome and transcriptome analyses identify the plant immunity systems that facilitate sesquiterpene and lignan biosynthesis in Syringa pinnatifolia Hemsl. BMC Plant Biol. 22, 132. doi: 10.1186/s12870-022-03537-5, PMID: 35317751 PMC8939180

[B10] Gao L. Yang X. Li Y. Wang Z. Wang S. Tan S. . (2021). Curcumol inhibits KLF5-dependent angiogenesis by blocking the ROS/ERK signaling in liver sinusoidal endothelial cells. Life Sci. 264, 118696. doi: 10.1016/j.lfs.2020.118696, PMID: 33157090

[B11] Haas B. J. Papanicolaou A. Yassour M. Grabherr M. Blood P. D. Bowden J. . (2013). *De novo* transcript sequence reconstruction from RNA-seq using the Trinity platform for reference generation and analysis. Nat. Protoc. 8, 1494–1512. doi: 10.1038/nprot.2013.084, PMID: 23845962 PMC3875132

[B12] Huang L. M. Huang H. Chuang Y. C. Chen W. H. Wang C. N. Chen H. H. (2021). Evolution of terpene synthases in orchidaceae. Int. J. Mol. Sci. 22, 6947. doi: 10.3390/ijms22136947, PMID: 34203299 PMC8268431

[B13] Jia Q. Li G. Köllner T. G. Fu J. Chen X. Xiong W. . (2016). Nonseed plant Selaginella moellendorffii has both seed plant and microbial types of terpene synthases. Proc. Natl. Acad. Sci. United States America 113, 12328–12333. doi: 10.1073/pnas.1607973113, PMID: 22908266 PMC3437839

[B14] Jiang L. Chen S. Wang X. Sen L. Dong G. Song C. . (2024). An improved genome assembly of Chrysanthemum nankingense reveals expansion and functional diversification of terpene synthase gene family. BMC Genomics 25, 593. doi: 10.1186/s12864-024-10498-6, PMID: 38867153 PMC11170872

[B15] Jiang C. Fei X. Pan X. Huang H. Qi Y. Wang X. . (2021). Tissue-specific transcriptome and metabolome analyses reveal a gene module regulating the terpenoid biosynthesis in Curcuma wenyujin. Ind. Crops Products 170, 113758. doi: 10.1016/j.indcrop.2021.113758

[B16] Li Y. Chen F. Li Z. Li C. Zhang Y. (2016). Identification and functional characterization of sesquiterpene synthases from xanthium strumarium. Plant Cell Physiol. 57, 630–641. doi: 10.1093/pcp/pcw019, PMID: 26858282

[B17] Li X. J. Liang L. Shi H. X. Sun X. P. Wang J. Zhang L. S. (2017). Neuroprotective effects of curdione against focal cerebral ischemia reperfusion injury in rats. Neuropsychiatr. Dis. Treat 13, 1733–1740. doi: 10.2147/NDT.S139362, PMID: 28721054 PMC5501624

[B18] Lin M. Jian J. B. Zhou Z. Q. Chen C. H. Wang W. Xiong H. . (2022). Chromosome-level genome of Entada phaseoloides provides insights into genome evolution and biosynthesis of triterpenoid saponins. Mol. Ecol. Resour. 22, 3049–3067. doi: 10.1111/1755-0998.13662, PMID: 35661414

[B19] Liu C. Cheng Y. Zhang H. Deng X. Chen F. Xu J. (2012). Volatile constituents of wild citrus mangshanyegan (Citrus nobilis lauriro) peel oil. J. Agric. Food Chem. 60, 2617. doi: 10.1021/jf2039197, PMID: 22352344

[B20] Liu Y. Wang W. Fang B. Ma F. Zheng Q. Deng P. . (2013). Anti-tumor effect of germacrone on human hepatoma cell lines through inducing G2/M cell cycle arrest and promoting apoptosis. Eur. J. Pharmacol. 698, 95–102. doi: 10.1016/j.ejphar.2012.10.013, PMID: 23117090

[B21] Mistry J. Chuguransky S. Williams L. Qureshi M. Salazar G. A. Sonnhammer E. L. L. . (2021). Pfam: The protein families database in 2021. Nucleic Acids Res. 49, D412–D419. doi: 10.1093/nar/gkaa913, PMID: 33125078 PMC7779014

[B22] Parker M. T. Zhong Y. Dai X. Wang S. Zhao P. (2014). Comparative genomic and transcriptomic analysis of terpene synthases in Arabidopsis and Medicago. Iet Syst. Biol. 8, 146–153. doi: 10.1049/iet-syb.2013.0032, PMID: 25075527 PMC8687412

[B23] Pertea M. Pertea G. M. Antonescu C. M. Chang T. C. Mendell J. T. Salzberg S. L. (2015). StringTie enables improved reconstruction of a transcriptome from RNA-seq reads. Nat. Biotechnol. 33, 290–295. doi: 10.1038/nbt.3122, PMID: 25690850 PMC4643835

[B24] Qu X. Hu S. Li T. Zhang J. Wang B. Liu C. (2022). Metabolomics Analysis Reveals the Differences Between Bupleurum chinense DC. and Bupleurum scorzonerifolium Willd. Front. Plant Sci. 13. doi: 10.3389/fpls.2022.933849, PMID: 35909726 PMC9328751

[B25] Robinson M. D. McCarthy D. J. Smyth G. K. (2010). edgeR: a Bioconductor package for differential expression analysis of digital gene expression data. Bioinformatics 26, 139–140. doi: 10.1093/bioinformatics/btp616, PMID: 19910308 PMC2796818

[B26] Ruan Q. Wang J. Xiao C. Yang Y. Luo E. Sheng M. . (2021). Differential transcriptome analysis of genes associated with the rhizome growth and sesquiterpene biosynthesis in Atractylodes macrocephala. Ind. Crops Products 173, 114141. doi: 10.1016/j.indcrop.2021.114141

[B27] Shang J. Tian J. Cheng H. Yan Q. Li L. Jamal A. . (2020). The chromosome-level wintersweet (Chimonanthus praecox) genome provides insights into floral scent biosynthesis and flowering in winter. Genome Biol. 21, 200. doi: 10.1186/s13059-020-02088-y, PMID: 32778152 PMC7419205

[B28] Sun J. Cui G. Ma X. Zhan Z. Ma Y. Teng Z. . (2019). An integrated strategy to identify genes responsible for sesquiterpene biosynthesis in turmeric. Plant Mol. Biol. 101, 221–234. doi: 10.1007/s11103-019-00892-0, PMID: 31203559

[B29] Wang J. Chitsaz F. Derbyshire M. K. Gonzales N. R. Gwadz M. Lu S. . (2023). The conserved domain database in 2023. Nucleic Acids Res. 51, D384–D388. doi: 10.1093/nar/gkac1096, PMID: 36477806 PMC9825596

[B30] Wang J. Li X. M. Bai Z. Chi B. X. Wei Y. Chen X. (2018). Curcumol induces cell cycle arrest in colon cancer cells via reactive oxygen species and Akt/ GSK3β/cyclin D1 pathway. J. Ethnopharmacology 210, 1–9. doi: 10.1016/j.jep.2017.06.037, PMID: 28684297

[B31] Xi W. Jiang M. Y. Zhu L. L. Zeng X. M. Ju H. Yang Q. L. . (2025). OfWRKY33 binds to the promoter of key linalool synthase gene OfTPS7 to stimulte linalool synthesis in Osmanthus fragrans flowers. Horticulture Res. 12, uhaf155. doi: 10.1093/hr/uhaf155, PMID: 40861036 PMC12373639

[B32] Xu P. Mi Q. Zhang X. Zhang X. Yu M. Gao Y. . (2025). Dissection of transcriptome and metabolome insights into the polyphyllin biosynthesis in Paris. BMC Plant Biol. 25, 206. doi: 10.1186/s12870-025-06219-0, PMID: 39955498 PMC11829371

[B33] Yang P. Ling X. Y. Zhou X. F. Chen Y. X. Wang T. T. Lin X. J. . (2023). Comparing genomes of Fructus Amomi-producing species reveals genetic basis of volatile terpenoid divergence. Plant Physiol. 193, 1244–1262. doi: 10.1093/plphys/kiad400, PMID: 37427874

[B34] Yu Y. Guan J. Xu Y. Ren F. Zhang Z. Yan J. . (2021). Population-scale peach genome analyses unravel selection patterns and biochemical basis underlying fruit flavor. Nat. Commun. 12, 3604. doi: 10.1038/s41467-021-23879-2, PMID: 34127667 PMC8203738

[B35] Yu N. Yang J. C. Yin G. T. Li R. S. Zou W. T. (2018). Transcriptome analysis of oleoresin-producing tree sindora glabra and characterization of sesquiterpene synthases. Front. Plant Sci. 9. doi: 10.3389/fpls.2018.01619, PMID: 30515178 PMC6256070

[B36] Yu Z. Zhao C. Zhang G. Teixeira Da Silva J. A. Duan J. (2020). Genome-wide identification and expression profile of TPS gene family in dendrobium officinale and the role of doTPS10 in linalool biosynthesis. Int. J. Mol. Sci. 21, 5419. doi: 10.3390/ijms21155419, PMID: 32751445 PMC7432446

[B37] Yuan H. Cao G. Hou X. Huang M. Du P. Tan T. . (2022). Development of a widely targeted volatilomics method for profiling volatilomes in plants. Mol. Plant 15, 189–202. doi: 10.1016/j.molp.2021.09.003, PMID: 34509640

[B38] Zeng J. Dai P. Ren L. Song B. Chen X. Wang X. . (2012). Apoptosis-induced anti-tumor effect of Curcuma kwangsiensis polysaccharides against human nasopharyngeal carcinoma cells. Carbohydr. Polymers 89, 1067–1072. doi: 10.1016/j.carbpol.2012.03.064, PMID: 24750915

[B39] Zhang C. Cao Y. Lin H. Wang Y. Wan X. Feng L. . (2025). Identification of candidate genes in sesquiterpenoid biosynthesis of Atractylodes lancea through combined metabolomic and transcriptomic analysis. Plant Physiol. Biochem. 224, 109822. doi: 10.1016/j.plaphy.2025.109822, PMID: 40239246

[B40] Zhou G. L. Li Y. Pei F. Gong T. Chen T. J. Chen J. J. . (2022). Chromosome-scale genome assembly of Rhododendron molle provides insights into its evolution and terpenoid biosynthesis. BMC Plant Biol. 22, 342. doi: 10.1186/s12870-022-03720-8, PMID: 35836128 PMC9284817

[B41] Zhou Y. Xie M. Song Y. Wang W. Zhao H. Tian Y. . (2016). Two traditional chinese medicines curcumae radix and curcumae rhizoma: an ethnopharmacology, phytochemistry, and pharmacology review. Evidence-Based Complementary Altern. Med. 2016, 4973128. doi: 10.1155/2016/4973128, PMID: 27057197 PMC4775794

[B42] Zhou Y. Yao L. Xie Y. Huang B. Li Y. Huang X. . (2025). Metabolic and transcriptional analysis of tuber expansion in Curcuma kwangsiensis. Sci. Rep. 15, 1588. doi: 10.1038/s41598-024-84763-9, PMID: 39794375 PMC11724066

[B43] Zhu X. Quan Y. Y. Yin Z. J. Li M. Wang T. Zheng L. Y. . (2023). Sources, morphology, phytochemistry, pharmacology of Curcumae Longae Rhizoma, Curcumae Radix, and Curcumae Rhizoma: a review of the literature. Front. Pharmacol. 14. doi: 10.3389/fphar.2023.1229963, PMID: 37719857 PMC10500466

