# Supplementary material for: Integration of metabolomics and transcriptomics unravels the identification of TPS gene family and functional characterization of a sesquiterpenoid synthesis gene in Curcuma kwangsiensis
Source: Front Plant Sci. 2025 Dec 3;16:1703946. doi: 10.3389/fpls.2025.1703946 (PMC12708598; doi:10.3389/fpls.2025.1703946)
Supplement: Supplementary file 1 [file DataSheet1.docx]

Supplementary Material

# Supplementary Figures and Tables


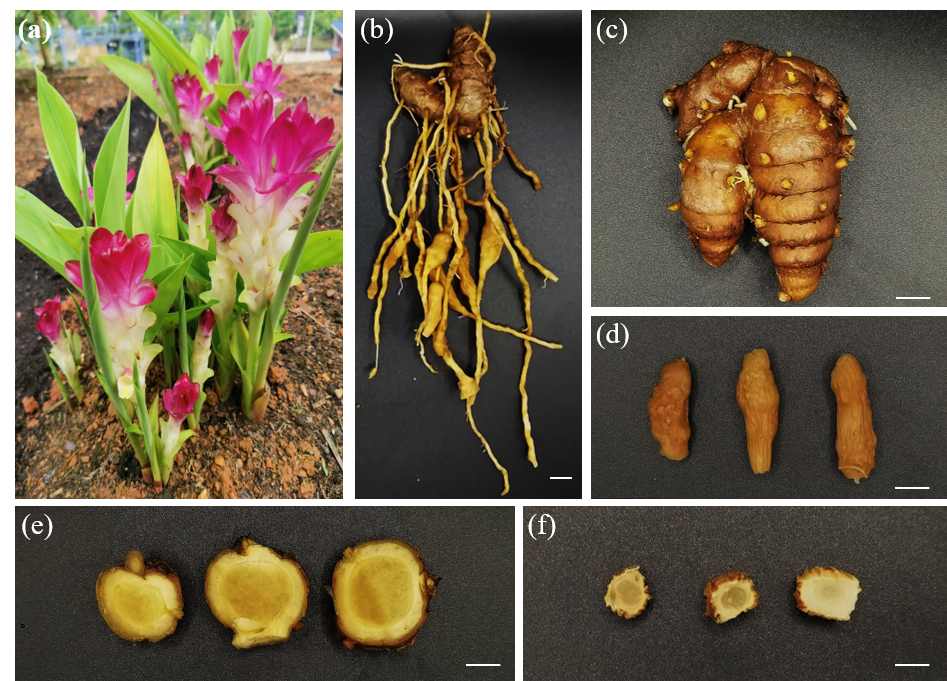


Supplementary Figure 1. The various plant parts of *Curcuma kwangsiensis*. The whole plant (a), underground part (b), tuber (c), rhizome (d), cross-cut of tuber (e), and cross-cut of rhizome (f). Each diagram is labeled according to the specific plant part it represents. The scale is indicated by a white horizontal line measuring 1cm.

Table S1 Functional annotation results statistics

| Database | Ref_gene |
| --- | --- |
| Total | 153,831(100.00%) |
| NR | 69,001 (44.86%) |
| GO | 58,910 (38.30%) |
| KO | 38,343 (24.93%) |
| KOG | 23,919 (15.55%) |
| Swiss-Prot | 58,143 (37.80%) |
| Unannotated | 81,411 (52.92%) |

Table S2 Primer information for qRT-PCR

| Gene | Primer（5’-3’） |
| --- | --- |
| *CkTPS1* | GGGAGATCATTTCGCCTCTACA  CGTTTAGATCTTTCCATGCTTTCT |
| *CkTPS6* | CCCACACTGGAGGAACATCTAA  TGCCACTTGATCCATTCCTACA |
| *CkTPS7* | GCATGGATCAAGTGGCATCA  GCTTGCTCTTTTTGTTGCTCAC |
| *CkTPS8* | GTGGAGCAATATGTGCCATCA  CTCTTCTCCCATGCCCAAAT |
| *CkTPS9* | TTGGGTATGGGAGAAGTGGC  ATTATACAGCAAGCTTCGACGG |
| *CkTPS10* | TAGTTCGTTAATCTCCTCTGGGTG  GCTTCTATGGGCTTGGGAAA |
| *CkTPS18* | AAGAGCACATGCGTGTTTCG  GCCACTCAAAACTACGCAAGAA |
| *CkTPS19* | CCAAGATGAACAGACCCGAAAT  CGTGTCACCATCAGCCAACA |
| *CkTPS22* | GAGCCGCCTTTAGCAACAGA  CATATCCTTCCACACCCCATTT |
| *CkTPS23* | TTGGAGCAAGAAAGAGACCATG  TGGATGGAGGCATTCCTTGT |

Table S3 Primer information for gene cloning and vector construction

| Gene | Primer（5’-3’） |
| --- | --- |
| *CkTPS6-F* | ATGTCACTTATGGATGACATATATGATAA  TTAAATAAGAATAGATTCAACCAACAAC |
| *CkTPS6-R* |  |
| *CkTPS7-F* | ATGGATTTAATTGATGCAATTCAGT  TTAAATAAGAATAGATTCAACCAACAGC |
| *CkTPS7-R* |  |
| *CkTPS10-F* | ATGGAGAAGCAATCACTAGCTCTT  TTATATGGGAAGAGGTTCAATCAA |
| *CkTPS10-R* |  |
| *pESC-URA-F* | *CTAAAGGGGGGATAGT* |
| *pESC-URA-R* | *TCATCCTTGTAATCCATCGAT* |

Table S4 Information of terpenoid differential metabolites in rhizome and tuber of *Curcuma kwangsiensis*

| No. | metabolite | quantitative value | | | LE vs EZ | | LE vs YJ | |
| --- | --- | --- | --- | --- | --- | --- | --- | --- |
|  |  | LE | EZ | YJ | VIP value | P-value | VIP value | P-value |
|  | (R)-Limonene oxide | 3.34E+03 | 7.15E+04 | 3.94E+04 | 1.03 | 0.0059 | 1.05 | 0.0057 |
|  | Terpinolene | 6.46E+05 | 2.27E+07 | 4.30E+06 | 1.03 | 0.0005 | 1.06 | 0.0064 |
|  | D-Berbenone | 4.01E+03 | 7.32E+04 | 3.75E+04 | 1.03 | 0.0006 | 1.05 | 0.0131 |
|  | Eudesmol | 2.31E+06 | 3.55E+08 | 1.44E+08 | 1.03 | 0.0003 | 1.06 | 0.0033 |
|  | Phellandrene | 1.19E+05 | 1.32E+08 | 8.97E+06 | 1.03 | 0.0007 | 1.04 | 0.0017 |
|  | Citronellal | 3.23E+04 | 3.30E+06 | 1.62E+06 | 1.03 | 0.0008 | 1.06 | 0.0014 |
|  | β-Cyclocitral | 2.67E+04 | 8.20E+04 | 5.74E+04 | 1.02 | 0.0016 | 1.03 | 0.0009 |
|  | 2,6-Dimethyl-2,4,6-octatriene | 9.00E+00 | 1.43E+05 | 5.94E+04 | 1.03 | 0.0040 | 1.06 | 0.0161 |
|  | Limonene | 1.40E+07 | 9.28E+07 | 3.58E+07 | 1.03 | 0.0007 | 1.05 | 0.0059 |
|  | (R)-(-)-Carvone | 9.00E+00 | 6.17E+05 | 6.71E+05 | 1.03 | 0.0007 | 1.06 | 0.0004 |
|  | 2-Methyl-6-methyleneoctyl-2,7-dienone | 8.42E+04 | 3.27E+07 | 1.72E+07 | 1.03 | 0.0003 | 1.05 | 0.0011 |
|  | α-Terpineol | 4.32E+05 | 2.18E+07 | 7.99E+06 | 1.03 | 0.0005 | 1.06 | 0.0057 |
|  | 6,7-Dimethyl-1,2,3,5,8,8a-hexahydronaphthalene | 9.00E+00 | 1.52E+04 | 9.18E+03 | 1.03 | 0.0022 | 1.06 | 0.0119 |
|  | Limonene epoxide | 5.62E+04 | 4.77E+06 | 2.42E+06 | 1.03 | 0.0004 | 1.06 | 0.0013 |
|  | o-cymene | 1.16E+06 | 2.58E+07 | 1.20E+07 | 1.03 | 0.0000 | 1.06 | 0.0034 |
|  | 4-Methyl-1-(1-methylethyl)-bicyclo[3.1.0]hex-3-one | 2.06E+04 | 6.89E+04 | 6.96E+04 | 1.01 | 0.0191 | 1.05 | 0.0062 |
|  | (-)-verbenone | 4.89E+04 | 1.15E+07 | 7.06E+05 | 1.03 | 0.0037 | 1.06 | 0.0045 |
|  | Camphor | 2.71E+05 | 1.35E+08 | 7.14E+07 | 1.02 | 0.0003 | 1.05 | 0.0012 |
|  | 2-(4-Methylcyclohexyl-2,4-dien-1-yl)prop-2-ol | 7.34E+04 | 3.93E+06 | 1.33E+06 | 1.03 | 0.0015 | 1.05 | 0.0056 |
|  | Trans-5-methyl-2-(1-methylethyl)-cyclohexanone | 9.00E+00 | 2.56E+05 | 1.20E+05 | 1.03 | 0.0012 | 1.06 | 0.0031 |
|  | (Z)-2,2-dimethyl-3-(3-methylpenta-2,4-diene-1-yl)ethyleneoxide | 9.00E+00 | 1.78E+05 | 6.64E+04 | 1.03 | 0.0009 | 1.06 | 0.0065 |
|  | (E)-β-ocimeneepoxide | 9.42E+04 | 9.96E+06 | 5.34E+06 | 1.03 | 0.0002 | 1.06 | 0.0009 |
|  | 3,6-Dihydro-4-methyl-2-(2-methyl-1-propenyl)-2H-pyran | 2.16E+04 | 1.43E+06 | 7.11E+05 | 1.02 | 0.0008 | 1.04 | 0.0007 |
|  | Menthol | 8.47E+05 | 1.66E+07 | 5.47E+06 | 1.03 | 0.0000 | 1.04 | 0.0043 |
|  | 4-Methyl-1-(1-methylethyl)-bicyclo(3.1.0)hex-3-en-2-one | 9.00E+00 | 2.25E+05 | 4.94E+04 | 1.03 | 0.0030 | 1.06 | 0.0109 |
|  | trans-(-)-Carvone-5,6-oxide | 1.83E+04 | 2.77E+05 | 1.11E+05 | 1.03 | 0.0013 | 1.05 | 0.0147 |
|  | 1,3,3-Trimethyl-2-oxabicyclo[2.2.2]octanone | 7.97E+03 | 1.06E+05 | 6.94E+04 | 1.03 | 0.0020 | 1.06 | 0.0043 |
|  | (R)-3,7-dimethyl-6-octen-6-ol | 1.05E+04 | 1.17E+05 | 1.00E+05 | 1.03 | 0.0026 | 1.05 | 0.0088 |
|  | 4-Isopropylbenzaldehyde | 1.70E+04 | 1.84E+05 | 1.12E+05 | 1.02 | 0.0111 | 1.03 | 0.0442 |
|  | α-Pinene | 6.87E+07 | 3.63E+08 | 1.67E+08 | 1.03 | 0.0006 | 1.06 | 0.0034 |
|  | 3,6,6-Trimethyl-bicyclo[3.1.1]hept-2-ene | 1.17E+06 | 6.05E+07 | 1.42E+08 | 1.03 | 0.0002 | 1.06 | 0.0023 |
|  | 3,7-Dimethyloctyl-1,6-diene | 8.56E+04 | 5.13E+06 | 1.17E+07 | 1.03 | 0.0006 | 1.06 | 0.0024 |
|  | 4-Methylene-1-(1-methylethyl)-bicyclo[3.1.0]hex-2-ene | 3.13E+05 | 1.91E+07 | 4.49E+07 | 1.03 | 0.0002 | 1.06 | 0.0027 |
|  | P-menthol-1,5,8-triene | 9.00E+00 | 1.42E+05 | 4.41E+04 | 1.03 | 0.0001 | 1.06 | 0.0005 |
|  | β-Phellandrene | 7.48E+07 | 4.35E+08 | 2.16E+08 | 1.03 | 0.0000 | 1.05 | 0.0016 |
|  | γ-Terpinene | 1.03E+06 | 1.15E+07 | 6.10E+06 | 1.03 | 0.0003 | 1.06 | 0.0065 |
|  | E,E-1,6-dimethyl-1,3,5,7-octene | 9.00E+00 | 3.17E+05 | 9.11E+04 | 1.03 | 0.0011 | 1.06 | 0.0077 |
|  | 1-Methyl-4-(1-methylvinyl)cyclohexanol | 3.20E+04 | 2.16E+06 | 1.04E+06 | 1.03 | 0.0008 | 1.06 | 0.0037 |
|  | (E,E)-2,6-dimethyl-2,4,6-octatriene | 3.92E+04 | 3.37E+05 | 1.80E+05 | 1.03 | 0.0001 | 1.06 | 0.0000 |
|  | 1-(1,4-dimethyl-3-cyclohexen-1-yl)-ethanone | 1.18E+05 | 4.30E+07 | 2.25E+07 | 1.03 | 0.0002 | 1.05 | 0.0011 |
|  | p-menth-8-en-1-ol,stereoisomer | 3.93E+04 | 9.53E+05 | 6.24E+05 | 1.02 | 0.0018 | 1.05 | 0.0009 |
|  | Camphol | 5.03E+05 | 1.55E+07 | 1.12E+07 | 1.03 | 0.0016 | 1.06 | 0.0009 |
|  | Trimethylbenzyl alcohol | 4.70E+04 | 1.52E+06 | 1.12E+07 | 1.03 | 0.0087 | 1.06 | 0.0009 |
|  | L-carveol | 9.00E+00 | 2.85E+05 | 1.08E+05 | 1.03 | 0.0009 | 1.06 | 0.0006 |
|  | (-)-trans-isopiperyl alcohol | 9.53E+03 | 3.00E+05 | 1.28E+05 | 1.03 | 0.0082 | 1.05 | 0.0080 |
|  | 5-methyl-2-(1-methylethylidene)-cyclohexanone | 9.00E+00 | 4.92E+05 | 5.04E+04 | 1.03 | 0.0028 | 1.06 | 0.0114 |
|  | (-)-Cis-carveol | 9.00E+00 | 4.08E+05 | 1.44E+05 | 1.03 | 0.0046 | 1.06 | 0.0143 |
|  | 3-Methyl-6-(1-methylethyl)-2-cyclohexen-1-one | 6.80E+04 | 9.18E+05 | 2.98E+05 | 1.03 | 0.0021 | 1.04 | 0.0107 |
|  | 3-Isopropyl-6-methyl-2-cyclohexen-1-one | 1.02E+05 | 9.81E+05 | 3.53E+05 | 1.03 | 0.0045 | 1.05 | 0.0099 |
|  | (1R,3aS,8aS)-7-isopropyl-1,4-dimethyl-1,2,3,3a,6,8a-hexahydroindene | 6.54E+04 | 1.30E+07 | 8.17E+06 | 1.03 | 0.0021 | 1.06 | 0.0047 |
|  | Β-Serinene | 8.71E+05 | 3.11E+07 | 2.75E+07 | 1.03 | 0.0001 | 1.06 | 0.0008 |
|  | (1S,2S,4R)-2-methyl-3-methylene-2-(4-methylpent-3-enyl)bicyclo[2.2.1]heptane | 9.00E+00 | 3.34E+06 | 2.11E+06 | 1.03 | 0.0009 | 1.06 | 0.0067 |
|  | α-Muurolene | 1.33E+05 | 6.61E+07 | 4.71E+07 | 1.03 | 0.0003 | 1.06 | 0.0021 |
|  | aR-Cedrene | 9.00E+00 | 5.00E+05 | 3.55E+05 | 1.03 | 0.0015 | 1.06 | 0.0072 |
|  | (Z,E)-3,7,11-trimethyl-2,6,10-dodecatrien-1-ol | 9.00E+00 | 1.69E+07 | 1.80E+07 | 1.03 | 0.0019 | 1.06 | 0.0002 |
|  | 5-Methyl-2-(1-methylethyl)-phenol acetate | 1.31E+04 | 4.47E+04 | 6.04E+04 | 1.01 | 0.0002 | 1.04 | 0.0060 |
|  | (3S,3aR,3bR,4S,7R,7aR)-4-isopropyl-3,7-dimethyloctahydro-1H-cyclopenta[1,3]cyclopropane[1,2]benzene-3-3-ol | 1.06E+05 | 8.45E+07 | 7.43E+07 | 1.03 | 0.0010 | 1.06 | 0.0021 |
|  | Cubenene | 1.03E+05 | 1.01E+07 | 6.84E+06 | 1.03 | 0.0005 | 1.06 | 0.0037 |
|  | (3aS,8aS)-6,8a-dimethyl-3-prop-2-ylidene-1,2,3a,4,5,8-hexahydromannulene | 9.00E+00 | 7.98E+06 | 6.97E+06 | 1.03 | 0.0002 | 1.06 | 0.0021 |
|  | (-)-α-Bisabolol | 9.00E+00 | 3.32E+07 | 3.72E+07 | 1.03 | 0.0018 | 1.06 | 0.0017 |
|  | [R-(R*,R*)]-α,4-dimethyl-α-(4-methyl-3-pentenyl)-3-cyclohexene-1-methanol | 9.00E+00 | 2.59E+05 | 2.61E+05 | 1.03 | 0.0034 | 1.06 | 0.0026 |
|  | (1S,4S,4aR)-1-isopropyl-4-methyl-7-methylene-1,2,3,4,4a,5,6,7-octahydronaphthalene | 5.55E+05 | 1.68E+07 | 1.16E+07 | 1.03 | 0.0002 | 1.06 | 0.0066 |
|  | (Z,E)-3,7,11-trimethyl-1,3,6,10-dodecene | 9.25E+04 | 1.35E+07 | 8.36E+06 | 1.03 | 0.0003 | 1.06 | 0.0046 |
|  | trans-Acorusene | 2.54E+05 | 1.56E+07 | 9.86E+06 | 1.03 | 0.0007 | 1.06 | 0.0066 |
|  | (4R,4aR)-4,4a-dimethyl-6-(prop-1-en-2-yl)-1,2,3,4,4a,7-hexahydronaphthalene | 2.89E+03 | 4.79E+05 | 3.01E+05 | 1.02 | 0.0027 | 1.05 | 0.0037 |
|  | α-Cadinene | 9.70E+04 | 6.61E+06 | 3.92E+06 | 1.03 | 0.0003 | 1.06 | 0.0028 |
|  | (1R,4aR)-7-methyl-4-methylene-1-prop-2-yl-1,2,3,5,6,8a-hexahydronaphthol | 3.73E+04 | 1.61E+06 | 1.18E+06 | 1.02 | 0.0006 | 1.05 | 0.0029 |
|  | Hinerol | 9.00E+00 | 5.66E+06 | 5.61E+06 | 1.03 | 0.0044 | 1.06 | 0.0012 |
|  | (2R-cis)-1,2,3,4,4a,5,6,7-octahydro-α.α.4a,8-tetramethyl-2-naphthylmethanol | 3.91E+03 | 2.40E+06 | 1.41E+06 | 1.03 | 0.0030 | 1.05 | 0.0022 |
|  | (4R,4aS,6S)-4,4a-dimethyl-6-(prop-1-en-2-yl)-1,2,3,4,4a,5,6,7-octahydronaphthalene | 1.23E+05 | 2.52E+07 | 1.34E+07 | 1.03 | 0.0004 | 1.06 | 0.0043 |
|  | β-Ionone | 9.00E+00 | 8.56E+05 | 4.63E+05 | 1.03 | 0.0003 | 1.06 | 0.0046 |
|  | 4-Isopropyl-6-methyl-1-methylene-1,2,3,4-tetrahydronaphthalene | 9.00E+00 | 1.05E+06 | 6.62E+05 | 1.03 | 0.0006 | 1.06 | 0.0034 |
|  | (4aR,8aR)-5,8a-dimethyl-3-propane-2-alkylidene-1,2,4,4a,7,8-hexahydronaphthalene | 7.26E+04 | 1.02E+07 | 5.59E+06 | 1.03 | 0.0003 | 1.06 | 0.0026 |
|  | Dehydrated sea cucumber terpene ketone | 4.53E+03 | 5.02E+04 | 6.36E+04 | 1.02 | 0.0136 | 1.06 | 0.0000 |
|  | Cubenol | 3.33E+04 | 1.01E+06 | 5.53E+05 | 1.03 | 0.0058 | 1.05 | 0.0031 |
|  | Zingiberene | 9.22E+04 | 3.73E+07 | 2.72E+07 | 1.03 | 0.0012 | 1.06 | 0.0029 |
|  | 3,6,6,9-Tetramethyl-4,4a,5,6,7,9a-hexahydro-1h-benzo[7]cycloene | 9.00E+00 | 3.94E+07 | 2.45E+07 | 1.03 | 0.0006 | 1.06 | 0.0045 |
|  | Α-Curcumene | 3.29E+06 | 1.83E+08 | 1.53E+08 | 1.03 | 0.0007 | 1.06 | 0.0024 |
|  | β-eucalyptol | 4.27E+03 | 1.68E+05 | 1.08E+05 | 1.03 | 0.0100 | 1.05 | 0.0038 |
|  | (+)-Longifolene | 9.00E+00 | 3.40E+05 | 1.29E+05 | 1.03 | 0.0002 | 1.06 | 0.0136 |
|  | α-Pinene | 5.99E+05 | 5.18E+06 | 2.80E+06 | 1.03 | 0.0002 | 1.05 | 0.0156 |
|  | 2,6,6,8-Tetramethyltricyclo[5.3.1.01,5]undec-9-ene | 5.14E+05 | 4.06E+06 | 2.21E+06 | 1.03 | 0.0002 | 1.05 | 0.0136 |
|  | Damasthone | 2.80E+03 | 1.74E+06 | 1.31E+06 | 1.03 | 0.0014 | 1.06 | 0.0057 |
|  | 3,7,11-trimethyl-1,6,10-dodecatrien-3-ol | 2.17E+04 | 3.00E+07 | 2.46E+07 | 1.03 | 0.0003 | 1.06 | 0.0012 |
|  | Ledol | 4.43E+04 | 1.48E+08 | 1.13E+08 | 1.03 | 0.0010 | 1.05 | 0.0028 |
|  | 6-Methyl-2-(4-methylcyclohex-3-en-1-yl)hept-1,5-diene-4-ol | 3.35E+04 | 4.24E+05 | 4.55E+05 | 1.01 | 0.0078 | 1.04 | 0.0000 |
|  | (1R,8aS)-1,6-dimethyl-4-(prop-2-yl)-1,2,3,7,8,8a-hexahydronaphthalene | 3.94E+05 | 5.39E+07 | 4.12E+07 | 1.03 | 0.0005 | 1.06 | 0.0015 |
|  | β-Guaiene | 1.12E+07 | 6.11E+08 | 5.39E+08 | 1.03 | 0.0001 | 1.06 | 0.0010 |
|  | (-)-Aristolene | 1.35E+05 | 6.25E+07 | 3.61E+07 | 1.03 | 0.0005 | 1.06 | 0.0051 |
|  | δ-Cadinene | 8.57E+05 | 3.68E+07 | 2.82E+07 | 1.03 | 0.0004 | 1.06 | 0.0041 |
|  | Isorutinenol | 4.51E+03 | 2.60E+06 | 2.34E+06 | 1.03 | 0.0054 | 1.06 | 0.0276 |
|  | (3R,3aS,8aR)-6,8a-dimethyl-3-propionyl-2-yl-1,2,3,4,5,8-hexahydroganju-3a-ol | 8.89E+05 | 5.51E+06 | 4.44E+06 | 1.02 | 0.0001 | 1.04 | 0.0022 |
|  | [3S-(3α,4α,5α)]-5-dimethyl-3-(1-methylvinyl)-3,4,4a,5,6,7-hexahydro-4a1(2H)-naphthone | 9.00E+00 | 7.51E+04 | 5.61E+04 | 1.03 | 0.0151 | 1.06 | 0.0017 |
|  | (1R,3aR,4aR,8aR)-1,4,4,6-tetramethyl-1,2,3,3a,4,4a,7,8-octahydrocyclopenta[1,4]cyclobuta[1,2]benzene | 1.81E+05 | 9.39E+07 | 6.94E+07 | 1.03 | 0.0014 | 1.06 | 0.0035 |
|  | trans-3a-cis-9a-1,2,3,3a,8,9,9a,9b-octahydro-4H-cyclopenta[def]phenanthrene | 9.00E+00 | 1.18E+06 | 8.49E+05 | 1.03 | 0.0032 | 1.06 | 0.0036 |
|  | α－Farnesene | 9.00E+00 | 2.14E+07 | 1.94E+07 | 1.03 | 0.0005 | 1.06 | 0.0018 |
|  | （-）-Eucalyptol | 9.00E+00 | 6.18E+04 | 3.21E+04 | 1.03 | 0.0006 | 1.06 | 0.0179 |
|  | [1R-(1α,3aβ,4α,8aβ,9S*)]-decahydro-1,5,5,8a-tetramethyl-1,4-methanoheptadien-9-ol | 4.93E+04 | 2.48E+06 | 1.81E+06 | 1.03 | 0.0004 | 1.06 | 0.0049 |
|  | Cedrenol | 2.63E+04 | 6.29E+05 | 7.40E+05 | 1.02 | 0.0056 | 1.05 | 0.0001 |
|  | (E)-5-(((1R,3R,6S)-2,3-dimethyltricyclo[2.2.1.02,6]heptyl-3-yl)-2-methyl-pent-2-ene | 9.00E+00 | 7.19E+06 | 5.19E+06 | 1.03 | 0.0003 | 1.06 | 0.0002 |
|  | 7-Acetyl-2-hydroxy-2-methyl-5-isopropylbicyclo[4.3.0]nonane | 9.00E+00 | 5.41E+05 | 3.40E+05 | 1.03 | 0.0015 | 1.06 | 0.0040 |
|  | Perilla alcohol | 1.47E+04 | 1.91E+05 | 3.20E+06 | 1.01 | 0.0000 | 1.05 | 0.0022 |
|  | 1-(1,5-dimethylhexyl)-4-methyl-benzene | 3.35E+04 | 1.48E+07 | 9.62E+06 | 1.03 | 0.0009 | 1.06 | 0.0057 |
|  | γ-Calacorene | 3.55E+04 | 7.40E+05 | 4.08E+05 | 1.03 | 0.0005 | 1.06 | 0.0064 |
|  | Biscuitene | 6.70E+05 | 1.38E+08 | 1.02E+08 | 1.03 | 0.0016 | 1.06 | 0.0048 |
|  | Cis-B-Farnesene | 1.27E+04 | 3.18E+06 | 2.06E+06 | 1.03 | 0.0017 | 1.06 | 0.0033 |
|  | (1R,3aS,8aS)-1,4,4,6-tetramethyl-1,2,3,3a,4,5,7,8-octahydrocyclopenta[c]pentene | 8.83E+03 | 1.46E+07 | 1.08E+07 | 1.03 | 0.0009 | 1.05 | 0.0073 |
|  | (E)-2,6-dimethyl-2,7-octadiene-1,6-diol | 8.83E+03 | 4.14E+05 | 3.25E+05 | 1.03 | 0.0007 | 1.06 | 0.0035 |
|  | (1R,2S,3S,6S,8S)-6-methyl-7-methylene-3-propane-2-yltricyclo[4.4.0.02,8]decane | 7.62E+04 | 8.35E+07 | 6.11E+07 | 1.03 | 0.0021 | 1.05 | 0.0036 |
|  | 3-Methyl-6-(1-methylethylidene)-2-cyclohexen-1-one | 1.11E+04 | 2.42E+07 | 1.85E+07 | 1.03 | 0.0006 | 1.05 | 0.0045 |
|  | Cis-Aromatic folic acid | 9.00E+00 | 3.46E+06 | 2.68E+06 | 1.03 | 0.0004 | 1.06 | 0.0043 |
|  | (-)-Α-Cubebene | 2.74E+05 | 6.10E+06 | 6.96E+06 | 1.03 | 0.0002 | 1.06 | 0.0036 |
|  | 1,1,7,7a-tetramethyl-1a,2,6,7,7a,7b-hexahydro-1H-cyclopropane[a]naphthalene | 3.61E+03 | 4.11E+05 | 3.02E+05 | 1.02 | 0.0018 | 1.05 | 0.0031 |
|  | [1aR-(1aα,3a,7bα)]-1a,2,3,3a,4,5,6,7b-octahydro-1,1,3a,7-tetramethyl-1H-cyclopropane[a]naphthalene | 7.73E+04 | 1.36E+08 | 9.56E+07 | 1.03 | 0.0017 | 1.05 | 0.0055 |
|  | （-）-Aristolene | 4.87E+04 | 1.92E+07 | 1.30E+07 | 1.03 | 0.0006 | 1.06 | 0.0051 |
|  | (-)-α-Gurjunene | 2.27E+04 | 3.96E+07 | 1.74E+07 | 1.02 | 0.0014 | 1.05 | 0.0098 |
|  | 2,6-Dimethyl-6-(4-methyl-3-pentenyl)-bicyclo[3.1.1]hept-2-ene | 2.82E+04 | 1.72E+07 | 1.13E+07 | 1.03 | 0.0044 | 1.06 | 0.0072 |
|  | Cis-α-bergamot | 7.60E+03 | 9.19E+06 | 4.31E+06 | 1.03 | 0.0009 | 1.06 | 0.0078 |
|  | 1,2,4a,5,6,8a-hexahydro-4,7-dimethyl-1-(1-methylethyl)naphthalene | 1.02E+05 | 6.09E+07 | 4.30E+07 | 1.03 | 0.0004 | 1.06 | 0.0025 |
|  | (1Z,6Z,8S)-1-methyl-5-methylene-8-prop-2-ylcyclodeca-1,6-diene | 9.96E+04 | 3.12E+07 | 2.09E+07 | 1.03 | 0.0011 | 1.06 | 0.0073 |
|  | γ-Selinene | 4.55E+04 | 6.30E+07 | 3.02E+07 | 1.03 | 0.0014 | 1.06 | 0.0080 |
|  | [1S-(1α,4β,5α)]-1,8-dimethyl-4-(1-methyltoluene)-spiro[4.5]decene | 1.22E+05 | 2.19E+07 | 1.17E+07 | 1.03 | 0.0006 | 1.06 | 0.0043 |
|  | Eremophilene | 1.78E+05 | 5.61E+07 | 3.36E+07 | 1.03 | 0.0005 | 1.06 | 0.0061 |
|  | (1S,4aR,7R)-1,4a-dimethyl-7-(propenyl)-1,2,3,4,4a,5,6,7-octahydronaphthalene | 8.64E+03 | 8.31E+05 | 1.56E+06 | 1.03 | 0.0037 | 1.06 | 0.0112 |
|  | (2R,4aS)-4a,8-Dimethyl-2-(prop-1-en-2-yl)-1,2,3,4,4a,5-hexahydronaphthalene | 1.63E+04 | 3.40E+06 | 1.77E+06 | 1.03 | 0.0006 | 1.06 | 0.0069 |
|  | (3R,8aR)-5,8a-dimethyl-3-(1-propenyl-2-yl)-1,2,3,7,8,8a-hexahydronaphthalene | 9.00E+00 | 1.15E+07 | 7.47E+06 | 1.03 | 0.0023 | 1.06 | 0.0041 |
|  | δ-Guaiene | 2.39E+06 | 1.10E+08 | 7.34E+07 | 1.03 | 0.0021 | 1.06 | 0.0024 |
|  | (1S,5S,6R)-6-methyl-2-methylene-6-(4-methylpent-3-en-1-yl)bicyclo[3.1.1]heptane | 1.37E+05 | 1.33E+07 | 8.24E+06 | 1.03 | 0.0011 | 1.06 | 0.0047 |
|  | (3R,3aR,3bR,4S,7R,7aR)-4-isopropyl-3,7-dimethyloctahydro-1H-cyclopenta[1,3]cyclopropyl[1,2]benzene-3-ol | 9.00E+00 | 7.14E+07 | 3.75E+07 | 1.03 | 0.0007 | 1.06 | 0.0082 |
|  | (5R,10R)-10-methyl-6-methylene-2-(propane-2-alkylidene)spiro[4.5]dec-7-ene | 2.33E+04 | 1.54E+07 | 8.38E+06 | 1.03 | 0.0007 | 1.06 | 0.0074 |
|  | 4a,8-dimethyl-2-(prop-1-en-2-yl)-1,2,3,4,4a,5,6,7-octahydronaphthalene | 3.58E+05 | 6.63E+07 | 4.64E+07 | 1.03 | 0.0007 | 1.06 | 0.0048 |
|  | Cis-α-Bisabolen | 5.04E+06 | 2.02E+08 | 1.37E+08 | 1.03 | 0.0018 | 1.06 | 0.0017 |
|  | 1-Methyl-4-(1,2,2-trimethylcyclopentyl)cyclohex-1,3-diene | 9.00E+00 | 6.92E+07 | 6.11E+07 | 1.03 | 0.0008 | 1.06 | 0.0020 |
|  | 4-(1,5-dimethyl-1,4-hexadiene)-1-methyl-cyclohexene | 1.32E+05 | 4.05E+06 | 3.22E+06 | 1.03 | 0.0002 | 1.06 | 0.0055 |
|  | Methylα-Ionone | 9.00E+00 | 1.35E+07 | 1.16E+07 | 1.03 | 0.0008 | 1.06 | 0.0029 |
|  | 7-octadiene bicyclo[4.1.0]heptane | 5.33E+04 | 1.89E+06 | 1.27E+06 | 1.03 | 0.0004 | 1.06 | 0.0048 |
|  | (E)-1-Methyl-4-(6-methylhept-5-en-2-ylidene)cyclohex-1-ene | 1.43E+04 | 3.95E+06 | 2.56E+06 | 1.03 | 0.0007 | 1.06 | 0.0031 |
|  | (1R,3aR,5aR,9aS)-1,4,4,7-tetramethyl-1,2,3,3a,4,5a,8,9-octahydrocyclopenta[c]benzofuran | 9.00E+00 | 1.11E+06 | 6.07E+05 | 1.03 | 0.0010 | 1.06 | 0.0057 |
|  | (1S,4R,7S,8R,11R,13R)-4,7,11-trimethyl-5-oxalatepentacyclo[5.4.2.01,8.04,13]tridecane | 9.00E+00 | 5.88E+05 | 3.34E+05 | 1.03 | 0.0004 | 1.06 | 0.0073 |
|  | (R)-1-methyl-4-(1,2,2-trimethylcyclopentyl)-benzene | 9.00E+00 | 2.38E+06 | 2.04E+06 | 1.03 | 0.0012 | 1.06 | 0.0016 |
|  | β-Sesquiterpenes | 4.52E+04 | 1.89E+06 | 1.35E+06 | 1.03 | 0.0017 | 1.06 | 0.0050 |
|  | 2-Methyl-6-(p-tolyl)hept-2-en-4-ol | 9.50E+05 | 4.85E+06 | 3.70E+06 | 1.01 | 0.0001 | 1.03 | 0.0024 |
|  | Guaiol | 1.41E+04 | 2.62E+06 | 1.39E+06 | 1.03 | 0.0016 | 1.05 | 0.0087 |
|  | 8-Isopropyl-2,5-dimethyltetralin | 6.31E+03 | 4.65E+06 | 2.74E+06 | 1.03 | 0.0014 | 1.06 | 0.0078 |
|  | [1S-(1α,3β,3aβ,4α,8aβ)]-decahydro-1,5,5,8a-tetramethyl-1,4-methylheptazen-1-ol | 8.34E+04 | 3.39E+05 | 2.57E+05 | 1.00 | 0.0005 | 1.01 | 0.0038 |
|  | Α-Didehydrocubebene | 3.61E+03 | 2.53E+05 | 1.77E+05 | 1.03 | 0.0022 | 1.06 | 0.0140 |
|  | Salvian-4(14)-en-1-one | 6.02E+03 | 9.77E+05 | 5.41E+05 | 1.03 | 0.0024 | 1.06 | 0.0104 |
|  | T-Oleyl alcohol | 4.62E+04 | 8.68E+05 | 4.89E+05 | 1.03 | 0.0075 | 1.05 | 0.0010 |
|  | Citronella acid ester | 9.00E+00 | 1.81E+06 | 8.42E+05 | 1.03 | 0.0027 | 1.06 | 0.0009 |
|  | β-Bisabolol | 3.21E+03 | 1.05E+05 | 1.32E+05 | 1.00 | 0.0004 | 1.03 | 0.0006 |
|  | α-Caryophyllenol | 1.82E+04 | 2.88E+07 | 2.26E+07 | 1.03 | 0.0007 | 1.06 | 0.0022 |
|  | (Z)-Epi-β-santalol | 9.00E+00 | 8.86E+06 | 9.68E+06 | 1.03 | 0.0021 | 1.06 | 0.0005 |
|  | （E)-farnesol | 9.00E+00 | 5.75E+05 | 4.12E+05 | 1.03 | 0.0021 | 1.06 | 0.0082 |
|  | lindazulene | 9.00E+00 | 2.86E+06 | 2.10E+06 | 1.03 | 0.0025 | 1.06 | 0.0070 |
|  | E,E-Farnesal | 9.00E+00 | 1.66E+05 | 9.18E+04 | 1.03 | 0.0020 | 1.06 | 0.0064 |
|  | [3S-[3α,6α(R*)]-tetrahydro-2,2,6-trimethyl-6-(4-methyl-3-cyclohexen-1-yl)-2H-pyran-3-ol | 1.43E+03 | 9.74E+05 | 6.02E+05 | 1.03 | 0.0016 | 1.06 | 0.0053 |
|  | 2-Methyl-6-[((1S)-4-methylcyclohex-3-en-1-yl]hept-2,6-diene-1-ol | 1.43E+03 | 9.74E+05 | 5.69E+04 | 1.03 | 0.0016 | 1.06 | 0.0133 |
|  | 2-[((2R,4aS)-4a,8-dimethyl-1,2,3,4,4a,5,6,7-octahydronaphthalene-2-yl]prop-2-ol | 9.00E+00 | 8.31E+05 | 3.45E+05 | 1.03 | 0.0022 | 1.06 | 0.0094 |
|  | 7-(2-Hydroxyprop-2-yl)-1,4a-dimethyldecalin-1-ol | 9.00E+00 | 2.46E+07 | 1.90E+07 | 1.03 | 0.0008 | 1.06 | 0.0034 |
|  | (4ar-cis)-5-dimethyl-3-(1-methylethylidene)-4,4a,5,6,7,8-hexahydro-4a-2(3H)-naphthone | 9.00E+00 | 3.33E+07 | 2.67E+07 | 1.03 | 0.0015 | 1.06 | 0.0052 |
|  | Cincumol | 9.00E+00 | 5.50E+05 | 3.29E+05 | 1.03 | 0.0010 | 1.06 | 0.0054 |
|  | Β-Elemene | 1.99E+05 | 1.77E+08 | 1.35E+08 | 1.03 | 0.0010 | 1.05 | 0.0026 |
|  | (E,E)-3,7-dimethyl-10-(1-methylethylidene)-3,7-cyclododecyl-1-one | 1.93E+04 | 1.90E+08 | 2.11E+08 | 1.03 | 0.0012 | 1.05 | 0.0006 |

Table S5 Metabolites enriched in key pathways of the sesquiterpene biosynthesis

| No. | Enriched metabolite name | Cas number | Classification of compounds | Up/down |
| --- | --- | --- | --- | --- |
| 1 | β-Selinene | 17066-67-0 | Terpenes | Up |
| 2 | cis-trans-Farnesol | 3790-71-4 | Terpenes | Up |
| 3 | Nerolidol | 7212-44-4 | Terpenes | Up |
| 4 | δ-Cadinene | 483-76-1 | Terpenes | Up |
| 5 | α-Farnesene | 502-61-4 | Terpenes | Up |
| 6 | trans-γ-Bisabolene | 53585-13-0 | Terpenes | Up |
| 7 | trans-Farnesol | 106-28-5 | Terpenes | Up |
| 8 | trans,trans-Farnesal | 502-67-0 | Terpenes | Up |
| 9 | (-)-Geosmin | 19700-21-1 | Arene | Down |

Table S6 Statistics of transcriptome data of *Curcuma kwangsiensis*

| Sample | | Clean Reads Pairs | | Clean base(bp) | | Q20(%) | Q30(%) | GC(%) |
| --- | --- | --- | --- | --- | --- | --- | --- | --- |
| GJ1 | 22,713,777 | | 6,814,133,100 | | 96.5 | | 89.7 | 47.8 |
| GJ2 | 24,935,858 | | 7,480,757,400 | | 95.7 | | 87.6 | 47.9 |
| GJ3 | 22,433,292 | | 6,729,987,600 | | 96.2 | | 88.9 | 47.9 |
| L1 | 24,380,211 | | 7,314,063,300 | | 96.0 | | 88.4 | 49.4 |
| L2 | 23,388,286 | | 7,016,485,800 | | 96.2 | | 88.9 | 49.2 |
| L3 | 24,851,878 | | 7,455,563,400 | | 96.4 | | 89.7 | 49.1 |
| YJ1 | 16,756,425 | | 5,026,927,500 | | 96.3 | | 89.3 | 43.8 |
| YJ2 | 23,092,171 | | 6,927,651,300 | | 96.5 | | 89.7 | 44.2 |
| YJ3 | 22,967,269 | | 6,890,180,700 | | 96.8 | | 90.5 | 44.3 |

Table S7 Information of high expressed DEGs in rhizome and tuber of *Curcuma kwangsiensis*

| Gene ID | L1 | L2 | L3 | GJ1 | GJ2 | GJ3 | YJ1 | YJ2 | YJ3 |
| --- | --- | --- | --- | --- | --- | --- | --- | --- | --- |
| TRINITY_DN83_c0_g1 | 216.98 | 218.3 | 227.04 | 1050.01 | 1156.34 | 1157.37 | 229.83 | 234.09 | 244.5 |
| TRINITY_DN66648_c0_g1 | 0 | 2.47 | 0 | 13.33 | 13.23 | 21.16 | 6.09 | 0 | 2.33 |
| TRINITY_DN10326_c0_g1 | 71.96 | 84.72 | 86.33 | 152.98 | 180.68 | 178.38 | 55.64 | 53.18 | 57.19 |
| TRINITY_DN10395_c0_g1 | 10.55 | 10 | 9.44 | 26.38 | 24.03 | 26.6 | 14.56 | 14.55 | 16.56 |
| TRINITY_DN21553_c0_g1 | 6.88 | 6.62 | 7.11 | 23.36 | 28.37 | 26.96 | 16.46 | 12.28 | 11 |
| TRINITY_DN11683_c0_g1 | 2.87 | 2.07 | 2.06 | 6.18 | 5.99 | 6.58 | 2.39 | 2.65 | 3.18 |
| TRINITY_DN83_c1_g1 | 45.79 | 42.42 | 45.52 | 85.15 | 142.46 | 128.03 | 74.7 | 80.23 | 57.69 |
| TRINITY_DN11906_c0_g1 | 162.2 | 162.21 | 158.45 | 513.06 | 519.58 | 525.62 | 205.24 | 208.96 | 211.88 |
| TRINITY_DN57852_c0_g1 | 3.42 | 5.88 | 6.96 | 33.5 | 38.44 | 35.7 | 5.19 | 5.14 | 8.08 |
| TRINITY_DN4631_c0_g1 | 39.82 | 36.65 | 39.57 | 112.71 | 112.73 | 116.1 | 62.76 | 65.23 | 65.45 |
| TRINITY_DN69379_c0_g1 | 3.95 | 6.02 | 4.69 | 9.96 | 9.86 | 15.17 | 6 | 4.61 | 4.98 |
| TRINITY_DN18315_c0_g1 | 11.87 | 12.18 | 15.82 | 32.64 | 29.67 | 27.85 | 11.43 | 8.29 | 11.18 |
| TRINITY_DN20913_c0_g1 | 0 | 0 | 0 | 17.59 | 19.65 | 17.27 | 5.08 | 0 | 1.94 |
| TRINITY_DN23840_c0_g1 | 16.56 | 11.28 | 17.65 | 28.95 | 32.46 | 33.67 | 7.49 | 10.37 | 7.59 |
| TRINITY_DN86616_c0_g1 | 40.62 | 41.87 | 43.35 | 106.61 | 124.19 | 115.54 | 95.39 | 93.49 | 102.44 |
| TRINITY_DN5802_c0_g1 | 38.92 | 39.99 | 34.28 | 237.51 | 234.8 | 239.18 | 89.04 | 91.31 | 92.82 |
| TRINITY_DN5195_c0_g1 | 1.02 | 0.97 | 1.14 | 4.4 | 4.1 | 3.61 | 2.12 | 2.07 | 2.62 |
| TRINITY_DN40116_c0_g1 | 0.04 | 0.03 | 0 | 0.9 | 1.27 | 0.98 | 6.95 | 6.25 | 6.07 |
| TRINITY_DN32619_c0_g1 | 9.03 | 10.33 | 10.07 | 20.62 | 20.12 | 21.09 | 31.96 | 32.53 | 32.84 |
| TRINITY_DN28564_c1_g1 | 0 | 0.15 | 0.14 | 3.04 | 4.79 | 3.93 | 4.88 | 4.85 | 6.14 |
| TRINITY_DN26764_c0_g1 | 58.81 | 56.52 | 51.43 | 201.9 | 189.38 | 186.74 | 111.73 | 103.82 | 113.26 |
| TRINITY_DN14065_c0_g1 | 4.88 | 4.13 | 3.21 | 6.54 | 5.73 | 5.77 | 11.41 | 11.5 | 11.59 |
| TRINITY_DN1461_c2_g1 | 26.14 | 38.66 | 27.04 | 86.85 | 93.7 | 72.27 | 125.14 | 112.14 | 119.13 |
| TRINITY_DN1503_c0_g1 | 0.58 | 0.8 | 0.76 | 2.19 | 1.89 | 1.99 | 1.96 | 2.43 | 1.65 |
| TRINITY_DN89051_c0_g1 | 0 | 0 | 0 | 0.96 | 0.81 | 0.87 | 0.45 | 0.26 | 0.05 |
| TRINITY_DN14871_c0_g3 | 0 | 0.21 | 0 | 24.27 | 24.88 | 25.11 | 38.6 | 34.03 | 39.1 |
| TRINITY_DN70490_c0_g1 | 0 | 0 | 0 | 8.89 | 9.07 | 12.08 | 8.59 | 6.45 | 13.58 |
| TRINITY_DN25010_c0_g1 | 0.25 | 0.18 | 0.33 | 0.36 | 1.09 | 0.86 | 0.87 | 0.64 | 1.1 |
| TRINITY_DN69327_c0_g1 | 0 | 0 | 0 | 0 | 0 | 0 | 3.25 | 2.37 | 2.89 |
| TRINITY_DN48453_c0_g1 | 0 | 0 | 0 | 0 | 0 | 0 | 54.18 | 53.45 | 51.68 |
| TRINITY_DN37097_c0_g1 | 0 | 0 | 0 | 0 | 0 | 0 | 5.92 | 5.4 | 4.26 |
| TRINITY_DN66261_c0_g1 | 0 | 0 | 0 | 0 | 0 | 0 | 0.93 | 0.83 | 1.13 |
| TRINITY_DN21913_c0_g1 | 0 | 0 | 0 | 0 | 0 | 0 | 32.34 | 31.46 | 31.91 |
| TRINITY_DN10756_c0_g1 | 0 | 0 | 0 | 0 | 0 | 0 | 4.79 | 5.28 | 5.25 |
| TRINITY_DN66261_c0_g2 | 0 | 0 | 0 | 0 | 0 | 0 | 1.56 | 1.19 | 1.17 |
| TRINITY_DN40067_c0_g1 | 0 | 0 | 0 | 0 | 0 | 0 | 35 | 36.1 | 30.87 |
| TRINITY_DN77407_c0_g1 | 0 | 0 | 0 | 0 | 0 | 0 | 72.25 | 72.06 | 71.03 |
| TRINITY_DN9295_c0_g1 | 0 | 0 | 0 | 0 | 0 | 0 | 95.14 | 106.15 | 96.79 |
| TRINITY_DN125967_c0_g1 | 0 | 0 | 0 | 0 | 0 | 0 | 2.33 | 1.06 | 1.75 |
| TRINITY_DN17478_c0_g1 | 0 | 0 | 0 | 0 | 0 | 0 | 36.59 | 34.87 | 33.78 |
| TRINITY_DN56046_c0_g2 | 0 | 0 | 0 | 0 | 0 | 0 | 1.35 | 1.73 | 0.99 |
| TRINITY_DN92999_c0_g1 | 0 | 0 | 0 | 0 | 0 | 0 | 0.35 | 1.32 | 0.78 |
| TRINITY_DN9857_c0_g1 | 0 | 0 | 0 | 0 | 0 | 0 | 62.95 | 47.01 | 45.76 |
| TRINITY_DN26552_c0_g1 | 0 | 0 | 0 | 0 | 0 | 0 | 26.04 | 26.4 | 25.04 |
| TRINITY_DN7772_c0_g1 | 0 | 0 | 0 | 0 | 0 | 0 | 15.75 | 18.74 | 16.08 |
| TRINITY_DN56046_c0_g1 | 0 | 0 | 0 | 0 | 0 | 0 | 1.89 | 2.61 | 0.94 |
| TRINITY_DN10042_c0_g1 | 0 | 0 | 0 | 0 | 0 | 0 | 49.41 | 50.59 | 50.39 |
| TRINITY_DN18206_c0_g1 | 0 | 0 | 0 | 0 | 0 | 0 | 46.39 | 44.76 | 43.44 |
| TRINITY_DN50562_c0_g1 | 0 | 0 | 0 | 0 | 0 | 0 | 1.21 | 1.25 | 1.18 |
| TRINITY_DN20912_c0_g1 | 0 | 0 | 0 | 0 | 0 | 0 | 27.99 | 28.44 | 27 |
| TRINITY_DN6845_c0_g1 | 0 | 0 | 0 | 0 | 0 | 0 | 24.97 | 25.74 | 22.47 |
| TRINITY_DN61656_c0_g1 | 0 | 0 | 0 | 0 | 0 | 0 | 2.67 | 4.27 | 3.66 |
| TRINITY_DN16170_c0_g1 | 0 | 0 | 0 | 0 | 0 | 0 | 72.97 | 68.68 | 68.24 |
| TRINITY_DN9211_c0_g1 | 0 | 0 | 0 | 0 | 0 | 0 | 60.91 | 63.28 | 61.84 |
| TRINITY_DN55248_c0_g1 | 0 | 0 | 0 | 0 | 0 | 0 | 1.57 | 1.22 | 1.51 |
| TRINITY_DN2480_c0_g1 | 2.38 | 2.43 | 2.38 | 2.08 | 2 | 2.02 | 7.46 | 6.02 | 6.03 |
| TRINITY_DN14120_c0_g1 | 0 | 0 | 0 | 0 | 0 | 0 | 58.23 | 57.99 | 52.67 |

Table S8 Genes annotated to secondary metabolic pathways related to terpene synthesis in *Curcuma kwangsiensis* transcriptome data

| metabolic pathway | Pathway ID | Number of genes enriched | |
| --- | --- | --- | --- |
|  |  | L vs GJ | L vs YJ |
| Terpenoid backbone biosynthesis | ko00900 | 24 | 39 |
| Sesquiterpenoid and triterpenoid biosynthesis | ko00909 | 3 | 4 |

Table S9 Physico-chemical properties of proteins encoded by TPS gene family of *Curcuma kwangsiensis*

| Gene | Gene ID | Numbers of amino acids | Theoretical isoelectric point(pi) | Instability coefficient | Liposolubility index | Subcellular localization prediction |
| --- | --- | --- | --- | --- | --- | --- |
| *CkTPS1* | TRINITY_DN14871_c1_g1 | 550 | 5.17 | 46.46 | 95.91 | Chloroplast |
| *CkTPS2* | TRINITY_DN14871_c1_g2 | 453 | 5.12 | 44.93 | 99.98 | Cytoplasm |
| *CkTPS3* | TRINITY_DN45438_c0_g1 | 488 | 5.57 | 49.10 | 90.14 | Chloroplast |
| *CkTPS4* | TRINITY_DN45438_c0_g2 | 555 | 5.95 | 47.33 | 87.87 | Cytoplasm |
| *CkTPS5* | TRINITY_DN45438_c0_g3 | 1131 | 8.67 | 50.95 | 84.72 | Extracellular ( secretory ) |
| *CkTPS6* | TRINITY_DN46424_c0_g1 | 251 | 5.32 | 44.81 | 99.28 | Cytoplasm |
| *CkTPS7* | TRINITY_DN46424_c0_g2 | 518 | 5.15 | 48.49 | 109.59 | Cytoplasm |
| *CkTPS8* | TRINITY_DN14871_c0_g2 | 628 | 5.45 | 40.40 | 93.44 | Cytoplasm |
| *CkTPS9* | TRINITY_DN14871_c0_g2 | 523 | 5.24 | 32.22 | 100.63 | Cytoplasm |
| *CkTPS10* | TRINITY_DN14871_c0_g3 | 548 | 5.44 | 38.31 | 97.51 | Cytoplasm |
| *CkTPS11* | TRINITY_DN2820_c0_g1 | 842 | 5.72 | 43.28 | 87.10 | Chloroplast |
| *CkTPS12* | TRINITY_DN11682_c0_g1 | 1301 | 6.59 | 47.00 | 88.15 | Chloroplast |
| *CkTPS13* | TRINITY_DN31269_c0_g1 | 623 | 5.71 | 43.03 | 84.83 | Cytoplasm |
| *CkTPS14* | TRINITY_DN70458_c0_g1 | 612 | 5.46 | 45.52 | 90.42 | Chloroplast |
| *CkTPS15* | TRINITY_DN86870_c0_g1 | 765 | 5.96 | 49.98 | 87.83 | Chloroplast |
| *CkTPS16* | TRINITY_DN23850_c0_g1 | 274 | 4.80 | 41.61 | 95.73 | Cytoplasm |
| *CkTPS17* | TRINITY_DN34312_c0_g1 | 590 | 5.36 | 40.59 | 86.14 | Cytoplasm |
| *CkTPS18* | TRINITY_DN42518_c0_g1 | 550 | 5.09 | 40.20 | 90.02 | Chloroplast |
| *CkTPS19* | TRINITY_DN42518_c0_g2 | 533 | 4.91 | 41.06 | 89.96 | Chloroplast |
| *CkTPS20* | TRINITY_DN9972_c0_g1 | 1202 | 6.14 | 47.02 | 89.60 | Chloroplast |
| *CkTPS21* | TRINITY_DN46691_c0_g1 | 392 | 5.59 | 56.90 | 86.99 | Chloroplast |
| *CkTPS22* | TRINITY_DN1467_c0_g2 | 674 | 5.10 | 37.94 | 92.14 | Extracellular ( secretory ) |
| *CkTPS23* | TRINITY_DN1467_c0_g1 | 329 | 5.26 | 37.30 | 90.97 | Chloroplast |
| *CkTPS24* | TRINITY_DN2820_c1_g1 | 838 | 5.44 | 43.57 | 86.13 | Chloroplast |

Table S10 Sesquiterpene products catalysed by the CkTPS10 by GC-MS

| Compound Name | Retention Time（RT） | Molecular Formula | Cas# | Chemical formula |
| --- | --- | --- | --- | --- |
| α-Copaene | 31.83 | C15H24 | 3856-25-5 |  |
| Farnesol | 45.19 | C15H26O | 4602-84-0 |  |
